# Curcumin-Piperlongumine Hybrids with a Multitarget Profile Elicit Neuroprotection in In Vitro Models of Oxidative Stress and Hyperphosphorylation

**DOI:** 10.3390/antiox11010028

**Published:** 2021-12-24

**Authors:** Ángel Cores, Noelia Carmona-Zafra, Olmo Martín-Cámara, Juan Domingo Sánchez, Pablo Duarte, Mercedes Villacampa, Paloma Bermejo-Bescós, Sagrario Martín-Aragón, Rafael León, J. Carlos Menéndez

**Affiliations:** 1 Unidad de Química Orgánica y Farmacéutica, Departamento de Química en Ciencias Farmacéuticas, Facultad de Farmacia, Universidad Complutense, 28040 Madrid, Spain; acores@ucm.es (Á.C.); noecarmo@ucm.es (N.C.-Z.); olmomart@ucm.es (O.M.-C.); jdsanche@ucm.es (J.D.S.); mvsanz@ucm.es (M.V.); 2 Instituto de Química Médica, Consejo Superior de Investigaciones Científicas (IQM-CSIC), 28006 Madrid, Spain; pablo.duarte@uam.es; 3 Instituto Teófilo Hernando y Departamento de Bioquímica, Facultad de Medicina, Instituto de Investigaciones Biomédicas ‘Alberto Sols’ UAM-CSIC, Universidad Autónoma de Madrid, 28029 Madrid, Spain; 4 Departamento de Farmacología, Farmacognosia y Botánica, Facultad de Farmacia, Universidad Complutense, 28040 Madrid, Spain; bescos@ucm.es

**Keywords:** oxidative stress, Tau aggregation, neuroinflammation, neuroprotection

## Abstract

Curcumin shows a broad spectrum of activities of relevance in the treatment of Alzheimer’s disease (AD); however, it is poorly absorbed and is also chemically and metabolically unstable, leading to a very low oral bioavailability. A small library of hybrid compounds designed as curcumin analogues and incorporating the key structural fragment of piperlongumine, a natural neuroinflammation inhibitor, were synthesized by a two-step route that combines a three-component reaction between primary amines, β-ketoesters and α-haloesters and a base-promoted acylation with cinnamoyl chlorides. These compounds were predicted to have good oral absorption and CNS permeation, had good scavenging properties in the in vitro DPPH experiment and in a cellular assay based on the oxidation of dichlorofluorescin to a fluorescent species. The compounds showed low toxicity in two cellular models, were potent inductors of the Nrf2-ARE phase II antioxidant response, inhibited PHF6 peptide aggregation, closely related to Tau protein aggregation and were active against the LPS-induced inflammatory response. They also afforded neuroprotection against an oxidative insult induced by inhibition of the mitochondrial respiratory chain with the rotenone-oligomycin A combination and against Tau hyperphosphorylation induced by the phosphatase inhibitor okadaic acid. This multitarget pharmacological profile is highly promising in the development of treatments for AD and provides a good hit structure for future optimization efforts.

## 1. Introduction

Oxidative stress and protein misfolding are two major hallmarks common to several neurodegenerative diseases. While reactive oxygen species (ROS) play an important role as second messengers in moderate concentrations in healthy cells, their hyperproduction combined with the impairment of antioxidant defenses cause an imbalance between ROS production and quenching known as oxidative stress, which is correlated with cell damage and senescence processes [1].

In proteins, oxidative stress induces alterations that induce protein misfolding. During the ageing process, an increase in oxidative stress and the collapse of the proteostatic system cause an imbalance between the generation and degradation of misfolded proteins, leading to their accumulation and the formation of inclusion bodies of β-amyloid or Tau, which have been observed in several neurodegenerative diseases. Tau protein aggregation is associated with the generation of paired helical filaments (PHF) and neurofibrillary tangles (NFT), which are common hallmarks of Alzheimer’s disease [2]. Furthermore, there is growing evidence suggesting that, in AD patients, Tau deposits are more relevant to neurodegeneration and cognitive decline than amyloid β (Aβ) plaques [3]. Tau is highly relevant to other degenerative diseases, collectively known as tauopathies, and a clear connection has been established between oxidative stress and the onset and progression of these diseases [4]. Tau protein aggregation is associated with two six-residue fragments, namely PHF6 (275VQIINK280) and PHF6* (306VQIVYK311). These fragments have a partially hydrophobic character and tend to interact with a β-cross-linked structure, contributing to the formation of the core of the PHFs, while the rest of the protein remains disordered. Protein oligomers are able to interact with these hexapeptide sequences and are the cause of protein–protein association in the early stages of the aggregation process [5,6]. They also produce alterations in microtubule interactions, reflecting a close relationship between the pathological and physiological functions of Tau. It has been postulated that both fragments can act as sites of action for inhibitors because of the role they play in aggregation when interaction between β-sheet structures occurs, with PHF6 being the main nucleation site driving aggregation. In particular, fibrils formed in vitro by PHF6 are similar to those generated from full-length Tau [7].

Cells have developed adaptive barriers to remove ROS and decrease the cellular insults that they generate. The Nrf2 protein is regarded as the master regulator of redox homeostasis in cells, and its activation unleashes an effective response against oxidative stress and to toxicity due to electrophiles. Nrf2 is physiologically bound in the cytosol to Keap1, its negative regulator. The Keap1-Nrf2 complex is confined to the cytoplasm, where it binds to the E3 ligase, allowing Nrf2 ubiquitination and its subsequent proteasomal degradation. By this mechanism, under basal conditions, Nrf2 is constitutively synthesized and swiftly hydrolyzed by the proteasome. Electrophilic or oxidative stimuli induce a conformational change in Keap1 after oxidation or covalent binding to the Cys-155 residue in the “sensor” region of the protein [8]. This modification in Keap1 releases Nrf2, preventing its proteasomal degradation, allowing its translocation into the nucleus. Finally, after Nrf2 binding to the ARE sequences of DNA, the transcription of cytoprotective and antioxidant genes is increased.

Natural products have traditionally been used to inspire the development of active compounds after structural modifications to improve their properties. The rhizome of Curcuma longa, known as turmeric, is employed as a spice throughout the world and has long been used in herbal medicine [9]. Curcumin, its major component, is a multifaceted compound with a broad spectra of activities including antioxidant, anti-inflammatory [10,11] and antiaggregative properties [12] that make it a highly promising, multitarget anti-Alzheimer hit compound [13]. Its antioxidant activity is related to its two phenolic functional groups, which are able to scavenge ROS directly, and to the presence of two electrophilic positions that allow covalent binding to the Cys-151 residue of Keap1 [14]. This binding leads to Nrf2 translocation to the nucleus and the subsequent transcription of cytoprotective and anti-inflammatory proteins. Curcumin also acts as a protein missfolding inhibitor, and in particular it efficiently inhibits Tau aggregation [15] due to the reduction of the levels of Tau oligomer [16] by the interaction of the drug with the PHF6 segment [17].

Despite the interesting neuroprotective profile of curcumin, its poor oral bioavailability and blood–brain barrier permeability [18], associated in part to its low metabolic and chemical stability, hampers its use as a successful therapeutic agent. The degradation of curcumin mainly involves: (a) the reduction of its olefin and carbonyl moieties; (b) the retro-aldol mediated cleavage of its β-diketone core, catalyzed by several aldo–keto reductases; (c) its reaction with glutathione by a Michael addition; (d) the conjugation of its phenolic and secondary hydroxyl groups with glucuronic acid [19].

**Scheme 1:**
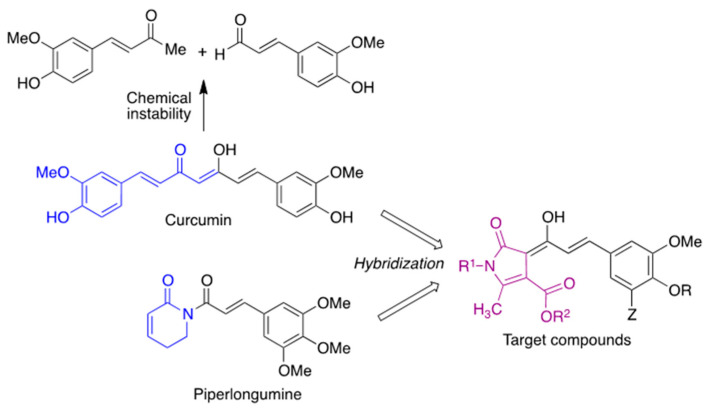
Compound design.

Against this backdrop, we envisioned curcumin analogues with their structure restricted by a heterocyclic core to increase its stability and maintain the pharmacological profile of curcumin while showing an improved bioavailability and introducing additional points of diversity in the structure. In order to guide the choice of substituents, we focused our attention on piperlongumine, an alkaloid extracted from the fruits of Piper longum that inhibits neuroinflammation and attenuates oxidative stress [20]. Structure–activity relationship studies have shown that the 3,4,5-trimethoxyphenyl fragment of this natural product increases its antioxidant activity [21], and a similar observation has been made on chalcones [22]. Thus, we designed compounds **2** by combining the key structural elements of both natural products (Scheme 1).

## 2. Materials and Methods

### 2.1. Chemistry

#### 2.1.1. General Experimental Details

The general experimental information was given in a previous article [23].

#### 2.1.2. General Procedure for the Synthesis of 2-Pyrrolin-5-one derivatives 1

The suitable β-ketoester (Equation (1)) and amine (Equation (1)), together with indium trichloride (5 mol%), were mixed in a microwave reaction vial and the resulting suspension was stirred at room temperature for 30 min. After verifying the completion of the enaminoester formation by TLC, excess amine was evaporated in vacuo. The suitable α-haloester (Equation (1)) was added and the mixture was heated under microwave irradiation (120 °C, 30 min). The cooled reaction mixture was diluted with CH_2_Cl_2_ (20 mL) and washed with water (2 × 5 mL). After drying over anhydrous sodium sulphate, the CH_2_Cl_2_ layer was and evaporated in vacuo. The residue was purified by chromatography on silica gel, using as mobile phase a gradient from hexane to 4:1 hexane-ethyl acetate. Compounds **1d** and **1e** have been previously described [24].

Methyl 2-methyl-5-oxo-1-(4-fluorophenethyl)-4,5-dihydropyrrole-3-carboxylate (**1a**).

Compound **1a** was obtained from 4-fluorophenethylamine (1 mmol), methyl acetoacetate (1 mmol) and ethyl bromoacetate (2 mmol), as a brown solid (156 mg, 56%); mp 74–75 °C; ^1^H NMR (250 MHz, CDCl_3_) δ 2.20 (t, *J =* 2.3 Hz, 3H), 2.84 (t, *J =* 7.3 Hz, 2H), 3.25 (q, *J =* 2.3 Hz, 2H), 3.75–3.64 (m, 5H), 7.02–6.94 (m, 2H), 7.15–7.07 (m, 2H); ^13^C NMR (63 MHz, CDCl_3_) δ 12.2, 34.5, 36.6, 42.1 (d, *J =* 1.2 Hz), 51.2, 103.1, 115.8 (d, *J =* 21.4 Hz, 2C), 130.4 (d, *J =* 8.0 Hz, 2C), 133.7 (d, *J =* 3.2 Hz), 154.3, 156.0, 161.9 (d, *J =* 245.8 Hz), 176.1; IR (neat, cm^−1^): 1213, 1622, 1687; elemental analysis (%) calcd. for C_15_H_16_FNO_3_: C 64.97, H 5.82, N 5.05; found: C 64.74, H 5.55, N 5.01.

Methyl 1-(4-fluorobenzyl)-2-methyl-5-oxo-4,5-dihydropyrrole-3-carboxylate (**1c**).

Compound **1c** was obtained from 4-fluorobenzylamine (1 mmol), methyl acetoacetate (1 mmol) and ethyl bromoacetate (1 mmol), as a brown solid (129 mg, 49%); mp 61–62 °C; ^1^H NMR (250 MHz, CDCl_3_) δ 2.34 (t, *J =* 2.4 Hz, 3H), 3.36 (q, *J =* 2.4 Hz, 2H), 3.72 (s, 3H), 4.72 (s, 2H), 7.07–6.95 (m, 3H), 7.21–7.11 (m, 2H); ^13^C NMR (63 MHz, CDCl_3_) δ 12.8, 36.7, 43.0, 51.3, 103.7, 116.0 (d, *J =* 21.7 Hz, 2C), 128.8 (d, *J =* 8.2 Hz, 2C), 132.4 (d, *J =* 3.3 Hz), 154.2, 162.4 (d, *J =* 246.8 Hz), 164.7, 176.1; IR (neat, cm^−1^): 1219, 1630, 1690; elemental analysis (%) calcd. for C_14_H_14_FNO_3_: C 63.87, H 5.36, N 5.32; found: C 62.53, H 5.18, N 5.34.

#### 2.1.3. Synthesis of Compounds **2**

A stirred solution of the suitable pyrrolinone derivative **1** (0.5 mmol) in anhydrous THF (5 mL), was treated dropwise at −78 °C with a 1.0 M solution of lithium hexamethyldisilazide (1.5 mL, 1.5 mmol) under an argon atmosphere and the resulting solution was stirred at −78 °C for 30 min. In parallel, oxalyl chloride (0.17 mL, 2 mmol) was added slowly to a suspension of the suitable cinnamic acid derivative (1 mmol) in anhydrous hexane (3.0 mL) containing anhydrous DMF (0.008 mL, 0.1 mmol) and this mixture was stirred at room temperature for 2 h. Removal of the solvent in vacuo furnished the crude cinnamoyl chloride derivative, which was immediately transferred via cannula into the pyrrolinone/LDA solution, and stirring at −78 °C was maintained for additional 30 min. After addition of saturated aqueous NH_4_Cl (5 mL), the reaction was left to warm to room temperature and was then acidified with 1M HCl. The aqueous solution was extracted with ethyl ether (3 × 20 mL). The combined ethereal layers were dried (anhydrous sodium sulphate) and evaporated in vacuo, and the residue was purified by chromatography on silica gel using as mobile phase a gradient from hexane to 1:1 hexane-ethyl acetate. Characterization data of new compounds follow, and copies of their spectra can be found in the Supporting Information (Supplementary Materials).

Methyl (*Z*)-1-(4-fluorophenethyl)-4-((*E*)-1-hydroxy-3-(3,4,5-trimethoxyphenyl)allylidene)-2-methyl-5-oxo-4,5-dihydro-1*H*-pyrrole-3-carboxylate (**2a**).

Compound **2a** was obtained from pyrrolinone **1a** (0.5 mmol) and 3,4,5-trimethoxycinnamoyl chloride (1 mmol), as an orange solid (100 mg, 40%); mp: 143–144 °C; ^1^H NMR (250 MHz, CDCl_3_) δ 2.16 (s, 3H), 2.92 (t, *J =* 7.1 Hz, 2H), 3.89–3.93 (m, 8H), 3.95 (s, 6H), 6.91 (s, 2H), 7.01 (t, *J =* 8.6 Hz, 2H), 7.09–7.20 (m, 2H), 7.64 (d, *J =* 15.8 Hz, 1H), 8.62 (dd, *J =* 15.8, 1.5 Hz, 1H), 14.00 (br d, *J =* 1.5 Hz, 1H); ^13^C NMR (63 MHz, CDCl_3_) δ 170.1, 167.7, 164.7, 162.2 (d, *J =* 245.4 Hz) 153.8 (2C), 148.8, 139.8, 139.7, 134.4 (d, *J =* 3.3 Hz), 132.4, 130.8 (d, *J =* 3.3 Hz, 2C), 120.7, 116.2 (d, *J =* 21.3 Hz, 2C), 105.7 (2C), 103.4, 102.3, 61.4, 56.6 (2C), 53.0, 42.6 (d, *J =* 1.2 Hz), 34.9, 13.9; IR (neat, cm^−1^): 1673.4, 1620.6, 1581.8, 1440.6, 1315.6; elemental analysis (%) calcd. for C_27_H_28_FNO_7_: C 65.18, H 5.67, N 2.82; found: C 64.87, H 5.69, N 2.88.

Methyl (*Z)*-1-butyl-4-((*E*)-1-hydroxy-3-(3,4,5-trimethoxyphenyl)allylidene)-2-methyl-5-oxo-4,5-dihydro-1*H*-pyrrole-3-carboxylate (**2b**).

Compound **2b** was obtained from pyrrolinone **1b** (0.5 mmol) and 3,4,5-trimethoxycinnamoyl chloride (1 mmol), as an orange solid (83 mg, 38%); mp: 128–129 °C; ^1^H NMR (250 MHz, CDCl_3_) δ 0.98 (t, *J =* 7.2 Hz, 3H), 1.34–1.46 (m, 2H), 1.52–1.66 (m, 2H), 2.52 (s, 3H), 3.66–3.76 (m, 2H), 3.87–3.99 (m, 12H), 6.90 (s, 2H), 7.61 (d, *J =* 15.8 Hz, 1H), 8.60 (dd, *J =* 15.8, 1.5 Hz, 1H), 13.97 (br d, *J =* 1.5 Hz, 1H); ^13^C NMR (63 MHz, CDCl_3_) δ 14.2, 14.3, 20.6, 32.0, 40.6, 53.0, 56.6 (2C), 61.4, 102.3, 103.6, 105.6 (2C), 120.6, 132.8, 139.4, 139.7, 149.0, 153.7 (2C), 164.4, 167.8, 170.1; IR (neat, cm^−1^): 1120.5, 1314.8, 1500.7, 1579.2, 1620.9, 1671.4; elemental analysis (%) calcd. for C_23_H_29_NO_7_: C 64.02, H 6.77, N 3.25; found: C 63.97, H 6.59, N 3.27.

(*Z*)-1-(4-fluorobenzyl)-4-((*E*)-1-hydroxy-3-(3,4,5-trimethoxyphenyl)allylidene)-2- methyl-5-oxo-4,5-dihydro-1*H*-pyrrole-3-carboxylate (**2c**).

Compound **2c** was obtained from pyrrolinone **1c** (0.5 mmol) and 3,4,5-trimethoxycinnamoyl chloride (1 mmol), as an orange solid (50 mg, 22%); mp: 169–170 °C; ^1^H NMR (250 MHz, CDCl_3_) δ 2.41 (s, 3H), 3.91 (s, 3H), 3.92–3.97 (m, 9H), 4.96 (s, 2H), 6.91 (s, 2H), 6.97–7.12 (m, 2H), 7.24–7.12 (m, 2H), 7.66 (d, *J =* 15.8 Hz, 1H), 8.65 (dd, *J =* 15.8, 1.6 Hz, 1H), 14.02 (br d, *J =* 1.6 Hz, 1H); ^13^C NMR (63 MHz, CDCl_3_) δ 14.6, 43.2, 53.1, 56.6 (2C), 61.4, 103.0, 103.2, 105.7 (2C), 116.2 (2C, d, *J =* 21.5 Hz), 120.5, 128.7 (2C, d, *J =* 8.2 Hz), 132.3, 132.9 (d, *J =* 3.2 Hz), 139.8, 139.9, 148.5, 153.7 (2C), 162.6, (d, *J =* 246.3 Hz), 165.1, 167.8, 170.1; IR (neat, cm^−1^): 1117.3, 1220.1, 1315.9, 1444.2, 1501.3, 1582.9, 1630.4, 1673.4; elemental analysis (%) calcd. for C_26_H_26_FNO_7_: C 64.54, H 5.42, N 2.90; found: C 64.48, H 5.22, N 2.97.

Ethyl (*Z*)-1-butyl-4-((*E*)-1-hydroxy-3-(3,4,5-trimethoxyphenyl)allylidene)-2-methyl-5-oxo-4,5-dihydro-1*H*-pyrrole-3-carboxylate (**2d**).

Compound **2d** was obtained from pyrrolinone **1d** (0.5 mmol) and 3,4,5-trimethoxycinnamoyl chloride (1 mmol), as an orange solid (87 mg, 39%); mp: 131–132 °C; ^1^H NMR (250 MHz, CDCl_3_) δ 0.97 (t, *J =* 7.0 Hz, 3H), 1.34–1.46 (m, 5H), 1.52–1.66 (m, 2H), 2.52 (s, 3H), 3.71 (t, *J =* 7.3 Hz, 2H), 3.89 (s, 3H), 3.93 (s, 6H), 4.39 (q, *J =* 7.0 Hz, 2H), 6.89 (s, 2H), 7.60 (d, *J =* 15.7 Hz, 1H), 8.60 (dd, *J =* 15.7, 1.5 Hz, 1H), 14.08 (br d, *J =* 1.5 Hz, 1H), ^13^C NMR (63 MHz, CDCl_3_) δ 14.2, 14.3, 14.8, 20.6, 32.0, 40.5, 56.6 (2C), 61.4, 62.3, 102.5, 103.7, 105.6 (2C), 120.7, 132.5, 139.3, 139.6, 148.9, 153.7 (2C), 164.4, 167.8, 169.7; IR (neat, cm^−1^): 1121.8, 1316.5, 1414.8, 1579.7, 1598.6, 1620.5, 1664.8; elemental analysis (%) calcd. for C_24_H_31_NO_7_: C 64.70, H 7.01, N 3.14; found: C 64.50, H 6.84, N 3.43.

Methyl (*Z*)-1-butyl-4-((*E*)-1-hydroxy-3-(4-hydroxy-3-methoxyphenyl)allylidene)-2-methyl-5-oxo-4,5-dihydro-1*H*-pyrrole-3-carboxylate (**2e**).

Compound **2e** was obtained from pyrrolinone **1b** (0.5 mmol) and feruloyl chloride (1 mmol), as an orange solid (30 mg, 15%); mp: 156–157 °C; ^1^H NMR (250 MHz, CDCl_3_) δ 0.94 (t, *J =* 7.2 Hz, 3H), 1.30–1.43 (m, 2H), 1.47–1.61 (m, 2H), 2.48 (s, 3H), 3.63–3.73 (m, 2H), 3.91 (s, 3H), 3.94 (s, 3H), 5.92 (br s, 1H), 6.89 (d, *J =* 8.7 Hz, 1H), 7.14–7.21 (m, 2H), 7.60 (d, *J =* 15.8 Hz, 1H), 8.55 (dd, *J =* 15.8, 1.6 Hz, 1H), 13.93 (br d, *J =* 1.6 Hz, 1H); ^13^C NMR (63 MHz, CDCl_3_) δ 13.9 (2C), 20.3, 31.7, 40.3, 52.6, 56.2, 102.0, 102.8, 109.7, 114.7, 118.6, 123.2, 129.2, 139.4, 146.8, 147.3, 148.2, 164.7, 167.4, 169.8; IR (neat, cm^−1^): 748.8, 978.3, 1117.3, 1284.7, 1322.3, 1571.6, 1587.0, 1619.9, 1647.8, 1698.1, 2870.6, 2630.3, 2955.8; elemental analysis (%) calcd. for C_21_H_25_NO_6_: C 65.10, H 6.50, N 3.62; found: C 64.93, H 6.38, N 3.93.

Methyl (*Z*)-1-benzyl-4-((*E*)-1-hydroxy-3-(3,4,5-trimethoxyphenyl)allylidene)-2-methyl-5-oxo-4,5-dihydro-1*H*-pyrrole-3-carboxylate (**2f**).

Compound **2f** was obtained from pyrrolinone **1e** (0.5 mmol) and 3,4,5-trimethoxycinnamoyl chloride (1 mmol); orange solid (72 mg, 31%); mp: 205 °C; ^1^H NMR (250 MHz, CDCl_3_) δ 2.40 (s, 3H), 3.91 (s, 3H), 3.92 (s, 3H), 3.94 (s, 6H), 5.00 (s, 2H), 6.91 (s, 2H), 7.18 (d, *J =* 6.8 Hz, 2H), 7.26–7.40 (m, 3H), 7.65 (d, *J =* 15.7 Hz, 1H), 8.67 (d, *J =* 15.7 Hz, 1H), 14.02 (br s, 1H); ^13^C NMR (63 MHz, CDCl_3_) δ 14.6, 43.8, 53.0, 56.6 (2C), 61.4, 102.8, 103.4, 105.6 (2C), 120.6, 126.9 (2C), 127.9, 129.3 (2C), 132.4, 137.1, 139.7, 149.1, 153.7 (2C), 164.8, 167.8, 170.2; IR (neat, cm^−1^): 1117.2, 1314.9, 1416.5, 1447.2, 1583.3, 1631.1 1674.1; elemental analysis (%) calcd. for C_26_H_27_NO_7_: C 67.09, H 5.85, N 3.01; found: C 66.84, H 5.68, N 3.29.

#### 2.1.4. Prediction of Physicochemical, ADME and CNS Permeability Properties

Physicochemical and ADME (absorption, distribution, metabolism, and excretion) property predictions for compounds **2** were carried out using the SwissADME platform [25] and the QikProp module of Schrödinger software [26]. Briefly, some physicochemical properties were calculated directly by introducing compound smiles in the SwissADME platform. For ADME predictions, ligand states were first generated at pH 7.4 using Epik [27] and they were then minimized using the LigPrep module [28]. Finally, ADME properties were calculated with QikProp [26]. Central nervous system (CNS) permeability for compounds **2** was evaluated using CNS multiparameter optimization (MPO) scoring algorithms [29,30,31].

#### 2.1.5. Antioxidant Capacity by the ORAC Assay

A FluoStar Optima plate reader (BMG LABTECH, GmbH, Offenburg, Germany) with 485 nm excitation and 520 nm emission filters [32,33] was used for this assay. 2,2′-Azobis-(amidinopropane) dihydrochloride (AAPH), trolox and fluorescein (FL) were purchased from Sigma-Aldrich and 75 mM phosphate buffer (pH 7.4) was used as the reaction medium. The solutions containing the antioxidant under assay (20 μL) and FL (120 μL at a 70 mM final concentration) were placed in a black 96-well microplate. Following pre-incubation at 37 °C for 15 min, a solution of AAPH (12 mM final concentration, 60 μL) was added in one portion using a multichannel pipette, resulting in a final reaction mixture 200 μL in volume. The microplate was placed in the reader and the fluorescence signal was measured at 1-min intervals for a total time of 90 min, while the reaction mixture was automatically shaken prior to each reading. Samples were measured at eight different concentrations in the 0.1–30 μM interval. A blank, containing FL + AAPH in phosphate buffer instead of the sample solution, and eight calibration trolox solutions (1–8 μM) were also measured in each assay. All the reaction mixtures were prepared in duplicate, and at least three independent assays were performed for each sample. Fluorescence vs. time curves, measuring the antioxidant response, were normalized with respect to the curve of the blank that had been measured in the same assay, and the area under the fluorescence decay curve (AUC) was calculated. The net AUC corresponding to a particular sample was obtained by subtracting from its AUC value the AUC corresponding to the blank. Regression equations between net AUC and antioxidant concentration were calculated for all samples. ORAC-FL values were expressed as trolox equivalents, which were calculated by using the standard curve calculated for each assay while taking the ORAC-FL value of trolox as 1.0.

#### 2.1.6. Anti-Oxidant Capacity by the 1,1-Diphenyl-2-picryl-hydrazyl (DPPH) Reduction Assay

The experimental conditions were modified from a previously described procedure [34]. Briefly, a solution of the compounds under assay (100 μL) at the desired final concentration (0.3, 3, 10, 30 and 100 μM) in 80/20 methanol/water were added, in a clear-bottom 96-well black plate, to a solution of DPPH in the same solvent mixture (100 μL, 100 μM final concentration) and the final solution was incubated in the dark for 30 min. The DPPH absorbances of blank (MeOH/water), control (DPPH 100 μM) and solutions containing compounds **2** plus DPPH were measured at 540 nm in a SpectroStar Nano plate-reader (BMG Labtech, Ortenberg, Germany), in duplicate experiments. The results are expressed as % of reduction in the absorbance (Abs) of the control after subtracting blank absorbance, as expressed in Equation (1) below:

(1) $$\%\mathrm{DPPH}\;\mathrm{reduction}=\frac{100-(Abs_{sample}-Abs_{blank})\times 100}{Abs_{control}}$$

#### 2.1.7. Computational Study of PHF6 Hexapeptide Aggregation

The structure of the PHF6 oligomer aggregate complexed with curcumin was obtained from https://people.mbi.ucla.edu/meytal/CoCrystalPaper/V6K-CUR/V6K-Cur.pdb. (accessed on 15 July 2021). Water molecules and curcumin were removed from the complex and the simplified model was then processed with AutoDockTools version 1.5.6 (Scripps Institute, La Jolla, CA, USA) to compute the Gasteiger charges and to obtain the AutoDock file. The grid box was determined by calculating the expected centre of the interaction area (x/y/z = −14.028/5.444/0.000) and its size was of x/y/z = 10/35/10 Å. Using this model, molecular docking was performed for the molecule of curcumin and compound **2d**, carried out by AutoDockVina [35], adjusting the number of conformations obtained to 9 and the exhaustiveness to 16.

Three molecular dynamics (MD) simulation were performed using Gromacs 2018.1 [36] and CHARMM36 [37,38,39,40,41] as force fields. The three systems studied included one with the oligomer alone (Apo-system) and two others with the oligomer complexed with a ligand, either curcumin (Cur-system) or compound **2d** (**2d**-system). Ligand conformations were obtained from the molecular docking calculation and further processing was carried out by UCSF Chimera 1.14, using the AMBER ff99bsc0 force field for the calculation of charges. Topologies and parameters of both the ligand and the enzyme were created with CgenFF [42]. The complex was solvated using the TIP3P force field and SPC model for water, and then minimized. A two-stage equilibration was performed by applying the NVT ensemble followed by the NPT ensemble for 50,000 2-fs steps. A 40 ns simulation was calculated for each ligand and conformation, with a time step of 2 ps and a cut-off of 1.0 nm. The PME method was employed to calculate the long-range electrostatic energies, using a four order cubic interpolation and a spaced grid of 0.16 nm. The temperature was established as 300 K, using a Berendsen thermostat with a coupling constant of 0.1 ps. The pressure was established at 1 bar and controlled with a Parrinello-Rahman barostat with a coupling constant of 2 ps at a compressibility of 4,5 × 10^−5^ bar^−1^. In order to evaluate the stability of the system during simulation, the root-mean-square displacement (RMSD), root-mean-square fluctuation (RMSF) and radius of gyration (Rg) for the protein and ligands were calculated for the conformations corresponding to any snapshot, compared with the conformation of the minimized state. The number of hydrogen bonds among the monomers themselves and between the oligomer and the ligands were also measured for any snapshot.

### 2.2. Biological Studies

#### 2.2.1. Culture of HEK293-Tau3R Cells

The stable cell line HEK293-Tau3R overexpressing Tau protein (HEK293-Tau3R, Tau 3R isoform) was kindly donated by Dr. Jesús Ávila (Center of Molecular Biology “Severo Ochoa”, Madrid, Spain). It was grown in Dulbecco’s Modified Eagle’s Medium (DMEM) (Lonza BioWhittaker, Porriño, Spain) supplemented with 10% fetal bovine serum (FBS), 2 mM glutamine, 1 mM sodium pyruvate and 0.2 mg/mL zeocin. Cultures were maintained at 37 °C in a humidified atmosphere with 5% CO_2_. Cells were digested with 0.25% trypsin during the logarithmic growth phase, and were then suspended in DMEM. This cell suspension was centrifuged (100 g, 5 min, rt) and the medium, still containing trypsin, was removed. Fresh medium was added to the cells and they were then seeded at appropriate densities on plates, according to the scale of each experiment. All measurements were carried out 24 h after seeding the cells. For treatments with the compounds under assay, HEK293-Tau3R cells were incubated for 24 h with reference compounds and curcumin analogues **2** in DMEM containing 1% FBS. Compound concentrations were selected taking into account the concentration-response data obtained in the cell viability test.

#### 2.2.2. Culture of SH-SY5Y Neuroblastoma Cells

SH-SY5Y cells were cultured in a 1:1 mixture of MEM and F12, supplemented with non-essential amino acids (0.5%), sodium pyruvate (0.5 mM), streptomycin (100 µg/mL), penicillin (100 units/mL) and 10% FBS. The cells were maintained at 37 °C in a humidified atmosphere formed by 95% air and 5% CO_2_ and were used for up to 14 passages.

#### 2.2.3. Culture of AREc32 Cells

AREc32 cells were kindly provided by Prof. Roland Wolf (University of Dundee, Dundee, UK). These cells were cultured at 37 °C in a 5% CO_2_ air atmosphere in DMEM medium containing glutamax and high-concentration glucose, supplemented with geneticin (0.8 mg/mL), 1% penicillin-streptomycin (10,000 units) and 10% FBS.

#### 2.2.4. Culture of BV2 Cells

BV2 mouse microglial cells, were cultured in RPMI medium, supplemented with penicillin (100 units/mL), streptomycin (100 µg/mL) and 10% FBS. Cells were maintained at 37 °C in a humidified atmosphere formed by 95% air and 5% CO_2_ and were used up to 16 passages.

#### 2.2.5. Cell Viability Studies in HEK293-Tau3R and SH-SY5Y Cells

Cells were plated in 96-well polystyrene plates at 20,000 cells per well (HEK293-Tau3R) or 60,000 cells per well (SH-SY5Y) densities and were incubated at 37 °C for 24 h to allow attachment of the cells. They were then incubated with the curcumin analogues **2** at concentrations of 5 and 10 µM (HEK293-Tau3R) or 100 µM (SH-SY5Y) for 24 h. In order to quantify cell viability, metabolic activity was measured by the ability of the cells to reduce 3-(4,5-dimethylthiazol-2-yl)-2,5-diphenyltetrazolium bromide (MTT, Sigma-Aldrich, Madrid, Spain) to its insoluble derivative formazan [43]. Absorbance measurements were taken at 550 nm using a SpectroStar Nano microplate reader (BMG LABTECH, Ortenberg, Baden-Württemberg, Germany). The mean of the optical density from the untreated cells (control cells) represents 100% of cell viability.

#### 2.2.6. Reactive Oxygen Species (ROS) Measurement in HEK293-Tau3R Cells

The dichlorofluorescin diacetate (DCFA-DA) probe was used to measure ROS generation [44] in HEK293-Tau3R cells. Cells were sub-cultured, and 24 h later they were treated with 10 μM DCFA-DA, which crosses the cell membrane and is processed by intracellular esterases to give dichlorofluorescin (DCFH). This compound was then oxidized by intracellular free radicals to form dichlorofluorescein (DCF), a highly fluorescent dye emitting in the green region. Compounds **2** were dissolved in DMSO and this stock solution was diluted in culture medium to various working concentrations so that the final DMSO concentration was below 0.002%. Curcumin analogues **2** (final concentration of 0.1–10 µM) were added to the cells and this was followed 30 min later by treatment with 200 μM hydrogen peroxide, a ROS generator. Fluorescence was measured after 60 min using a FLUOSTAR microplate reader (BMG LABTECH, Ortenberg, Baden-Württemberg, Germany) with the excitation filter set at 485 nm (bandwidth 5 nm) and the emission filter set at 520 nm (bandwidth 5 nm). The data thus obtained were presented as fluorescence arbitrary units (FAU).

#### 2.2.7. PHF6 Peptide Aggregation

This in vitro aggregation assay mimics the expected steps associated with Tau misfolding and aggregation in vivo. Tau aggregation is based on the transition to β-sheet structure, and was studied using the hexapeptide 306VQIVYK311 (PHF6), which in solution is capable of forming amyloid-like fibrils, with β-parallel cross-linked structure. The acetyl-PHF6 amide (01-H-8112, Cymit Química, Barcelona, Spain) peptide was dissolved in water at a concentration of 1 mM and before adding it to the reaction mixture it was sonicated for 2 min in order to monomerize it. Unlike other proteins with the ability to aggregate, Tau protein does not spontaneously form fibrils when incubated under physiological conditions. To facilitate the study of in vitro polymerization, inducing polyanionic molecules such as heparin are required. Therefore, the in vitro aggregation was triggered by addition of heparin (final concentration of 1 µM) and monitored continuously by thioflavin T (ThT) fluorescence in a 96 well microplate format.

#### 2.2.8. Thioflavin T Assay

Stock solutions of PHF6 were placed in 100 µL wells in a 96-well black plate and diluted so that the final concentrations were 100 µM of the peptide and 10 µM ThT in 50 mM phosphate buffer. In order to initiate peptide aggregation, heparin was added (final concentration of 1 µM) immediately prior to the experiment. Then, 10 μM concentrations of the molecules under assay, namely compounds **2**, curcumin (standard) or methylene blue (standard), were added separately to designated wells for one hour at room temperature to allow the fluorescent probe (ThT) to bind to the β-sheet structures thus generated. Control wells were supplemented with the same volume of phosphate buffer as test wells that contained the curcumin analogues **2**. From time zero, kinetic fluorescence data were collected at 37 °C for PHF6 in triplicate using a SPECTROstar microplate reader (BMG LABTECH, Ortenberg, Baden-Württemberg, Germany) and measurements were taken at 5-min intervals over 60 min for inhibition (or self-assembly), discounting the basal fluorescence of the target incubated under the same conditions. The excitation and emission wavelengths of ThT were 440 and 508 nm, respectively. Each set of ThT experiments was performed in triplicate and was repeated three times with similar observations. The results are represented as the average of three replicates (*n* = 3) and the error bars are indicated as the ± SEM.

#### 2.2.9. Nrf2 Induction Capacity

AREc32 cells were seeded in 96-well white plates (2 × 10^4^ cells/well) and incubated with compounds **2** (0.3, 3, 10 and 30 µM) in duplicate for 24 h. Each plate included non-treated cells as basal luciferase expression and *tert*-butylhydroquinone (TBHQ, 10 μM) as positive control. The Luciferase Assay System (Promega, Madrid, Spain) was employed, and measurements were taken in an Orion II microplate luminometer (Berthold, Germany). The observed increases in luciferase activity were normalized to basal conditions, which were taken as 1. The concentrations required to double the luciferase activity (CD values) were calculated from dose-response curves generated from graphical representation of luciferase fold induction vs. compound concentration, and were fitted by non-linear regression techniques and the data were interpolated to a value of 2.

#### 2.2.10. Neuroprotection Assays in SH-SY5Y Cells

SH-SY5Y human neuroblastoma cells were seeded in 96-well plates (6 × 10^5^ cells/well). To evaluate the neuroprotective effect of compounds **2**, the rotenone (30 μM) and oligomycin A (10 μM) (R/O) combination was employed as a model of oxidative stress and okadaic acid (OA, 20 nM) was employed as a model of Tau hyperphosphorylation. Cells were pre-incubated with compounds **2** (1 µM) for 24 h and treatments were then removed and cells were incubated for 24 h with the compounds (1 µM) in the presence of the R/O mixture (30/10 µM). When the experiments were finished, cell viability was assessed by the MTT method using a SPECTROstar Nano spectrophotometer (BMG Labtech, Ortenberg, Baden-Württemberg, Germany) to measure the absorbance at 570 nm. Data were normalized to cell death induced by the toxic stimuli (considered as 100% cell death).

#### 2.2.11. Nitrite Production Reduction Assay

BV2 microglial cells were treated with compounds at the 0.1, 0.3, 1 and 10 µM concentrations during 24 h in complete culture media. At this point, treatments were removed and cells were co-incubated with compounds **2** and LPS (100 ng/mL) for an additional period of 18 h. Each plate included also non-treated cells for basal nitrite production. Nitrite levels were determined using a modified Griess assay. In brief, samples (150 μL) were mixed with DAPSONE (75 μL) and NEDA (75 μL), and the mixtures were incubated at rt for 5 min. Absorbances were measured at 550 nm in a microplate reader using a SPECTROstar Nano instrument (BMG Labtech, Ortenberg, Baden-Württemberg, Germany). Basal nitrite production was considered 100% nitrite production and all data were normalized to the basal condition.

### 2.3. Statistical Analyses

All quantitative values are given as mean ± S.E.M. The IC_50_ and SC_50_ parameters were calculated by non-linear regression analysis from individual concentration-response curves using the GraphPad Prism software (San Diego, CA, USA). All measurements were conducted in at least three independent experiments and all assays were performed in performed in duplicate or triplicate, as specified for each experimental protocol. Data were analyzed using a one-way analysis of variance (ANOVA) followed by the Newman–Keuls post-hoc test. Statistical significance for all parameters was set at *p* < 0.05.

## 3. Results and Discussion

### 3.1. Prediction of Physicochemical, ADME and CNS Permeability Properties of Compounds **2**

The main physicochemical properties and ADME predictions for curcumin and compounds **2** are summarized in Table 1. Interestingly, our compounds showed in general a better profile than curcumin in terms of predicted Caco-2 cell and MDCK cell permeability. This is important for CNS drug design, taking into account that MDCK cells permeability has been found to be a useful predictor of the ability of a compound to cross the blood–brain barrier (BBB). In addition, compounds **2** also displayed high human oral absorption prediction, improving that of curcumin. This prediction takes into account the number of metabolites, number of rotatable bonds, logP, solubility and cell permeability, among other factors. These are promising results, bearing in mind that curcumin exhibits very poor bioavailability due to its poor absorption, besides other issues related to its poor stability [45].

We have also calculated CNS MPO scores for curcumin and compounds **2** as a CNS permeability prediction. This prediction is based on several physicochemical parameters as lipophilicity (cLogP), distribution coefficient at pH 7.4 (cLogD), molecular weight, topological polar surface area (tPSA), number of hydrogen-bond donors (HBD), and most basic center (pKa) [29]. The final CNS MPO score vary from 0–6 and its potential has been evaluated through studying a set of marketed CNS drugs and Pfizer CNS candidates, showing how this tool may guide the design of new molecules in order to increase the percentage of success when targeting the brain. Importantly, 74% of marketed CNS drugs displayed a CNS MPO score higher than 4.0, in comparison to 60% of the Pfizer CNS candidates [30]. Rankovic [31] developed a modified version of the CNS MPO algorithm (CNS MPO.v2), taking into account that the results obtained from a new set of compounds showed that most critical physicochemical properties in regard to CNS permeability were molecular size and hydrogen bonding capacity. This second version of the algorithm (CNS MPO.v2) is the one employed in this study and the results are summarized in Table 2. In general, compounds **2** exhibited improved CNS permeability in comparison with curcumin, with CNS MPO.v2 scores being higher than 4 in all cases and almost reaching 5 in the case of compound **2c**. These are encouraging results, since the above-mentioned analysis shows that marketed CNS drugs have CNS MPO scores in the same range. It is relevant to note that curcumin has shown good potential for the treatment of neurodegenerative diseases, but its poor BBB permeability is a major obstacle limiting its potential [46]. Thus, although there is evidence that curcumin is able to cross BBB, its presence in the mouse brain was weak and an increase in the number of administrations was required to achieve its CNS accumulation [47,48]. For this reason, it is of great interest for the development of novel curcumin-related compounds that show an improved CNS permeability and have therefore the potential to overcome this problem. All our compounds gave a score above the 4.0 threshold, which is typical for considering that a compound is able to reach CNS and their differences with curcumin are remarkable in most cases. The comparison between the best compound in this prediction (**2b**) with curcumin shows almost 1 unit of difference and represents a very significant ca. 20% improvement. Similarly, compounds **2d**–**f** show CNS MPO scores at least 0.5 units higher than curcumin.

### 3.2. Synthesis of Compounds **2**

**Scheme 2:**
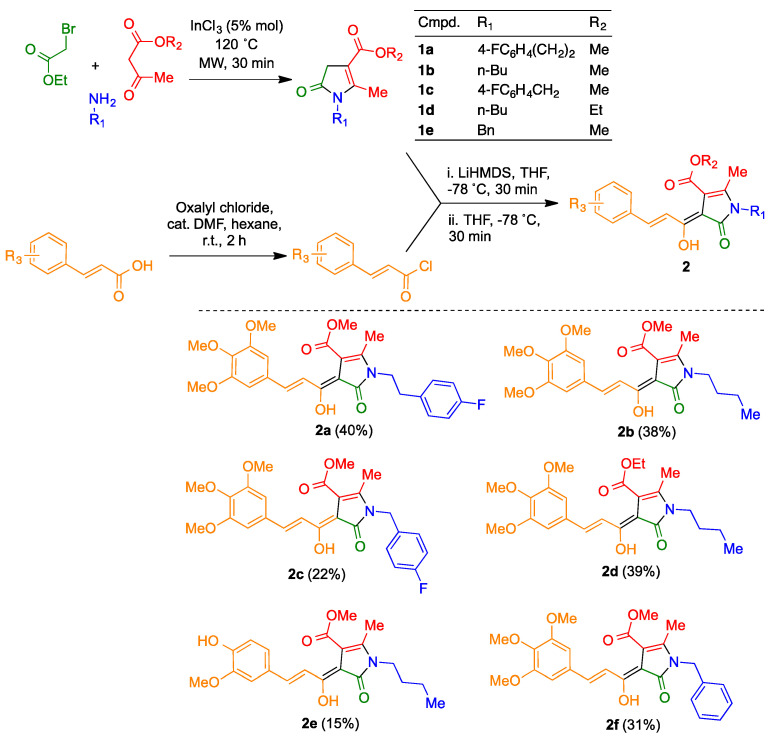
Synthesis of the curcumin/piperlongumine hybrids **2**.

The curcumin/piperlongumine hybrids **2a**–**f** were obtained by acylation of pyrrolinones **1**, prepared via a known three-component reaction between primary amines, β-ketoesters and α-bromoesters [23], with the suitable cinnamyl chloride derivative, using LiHMDS as a base (Scheme 2). Several attempts to use thionyl chloride for this transformation met with failure, and thus established adequate reaction conditions for the synthesis of the required acyl chlorides required some experimentation. Finally, the transformation could be performed with a mixture of oxalyl chloride and dimethylformamide [49] using as solvent hexane, which extracted the unstable cinnamyl chloride as it was formed.

### 3.3. In Vitro Characterization of the Antioxidant Capacity of Compounds **2**

Curcumin is a potent free radical scavenger able to trap oxygen and nitrogen-derived free radicals [50]. To evaluate the potential antioxidant capacity of our derivatives we firstly used the Oxygen Radical Absorbance Capacity (ORAC) assay, a method based on the generation of oxygen-derived free radicals using trolox (a vitamin-E analogue with potent antioxidant capacity) as reference. Curcumin was included as a control and for comparative purposes, and the results are depicted in Table 3. Curcumin showed a potent antioxidant profile, being a 3.88-fold more potent scavenger than trolox, in agreement with previously reported results [51]. This property of curcumin has been related to the presence of free -OH substituents at aromatic rings. Accordingly, derivatives **2a**–**c** and **2f**, bearing only methoxy substituents, showed a poor scavenger activity compared to the natural product. On the other hand, the phenolic derivative **2e** showed the best scavenger activity of the library, being 1.73-fold more potent than trolox to trap oxygen-derived free radicals. Surprisingly, derivative **2d**, the only one bearing a ethyl ester moiety, also showed a high capacity to trap free radicals, being 1.63-fold more potent than trolox and 3.13-fold more potent than its methyl ester analogue **2b**. Modification of the *N*-substituent did not affect scavenger capacity.

In order to obtain further insights into the antioxidant properties of our compounds, we tested their capacity to scavenge the 1,1-diphenyl-2-picrylhydrazyl (DPPH) free radical [52]. Compounds **2** were tested at increasing concentrations (0.3, 3, 10, 30 and 100 μM) to calculate the concentration of compound able to scavenge 50% of DPPH-derived free radicals (SC_50_). Ascorbic acid was included as a positive control and reference. Gratifyingly, compounds **2a-f** showed potent scavenger effect with SC_50_ values ranging from 8.86 ± 0.45 μM (**2e**) to SC_50_ = 56.9 ± 1.18 μM (**2c**) (Table 3), being compounds **2b** (SC_50_ = 10.8 ± 0.06 μM), **2d** (SC_50_ = 14.2 ± 2.15 μM) and **2e** (SC_50_ = 8.87 ± 0.45 μM) more potent than ascorbic acid (SC_50_ = 17.2 ± 1.14 μM). Considering the substitution pattern, *N*-arylalkyl derivatives showed, in all cases, lower DPPH scavenger capacity, with SC_50_ values higher than 20 μM. Among them, inclusion of an additional methylene group at the *N*-substituent of the fluorobenzyl group of compound **2a** (SC_50_ = 20.6 ± 2.17 μM) improved its potency 2.76-fold with respect to compound **2c** (SC_50_ = 56.9 ± 1.18 μM). This result indicates the influence of the electronic effect in the scavenger activity of our derivatives. Considering alkyl derivatives, similarly to ORAC results, compound **2e** (−OH, −OMe derivative) showed the highest antioxidant capacity (SC_50_ = 8.87 ± 0.45 μM), which can be related to the presence of the free hydroxy group at the aromatic ring at the aryl-allylidene moiety.

### 3.4. Biological Evaluation of Curcumin Derivatives

#### 3.4.1. Cytotoxicity Evaluation in the HEK293-Tau3R and SH-SY5Y Cell Lines

**Figure 1:**
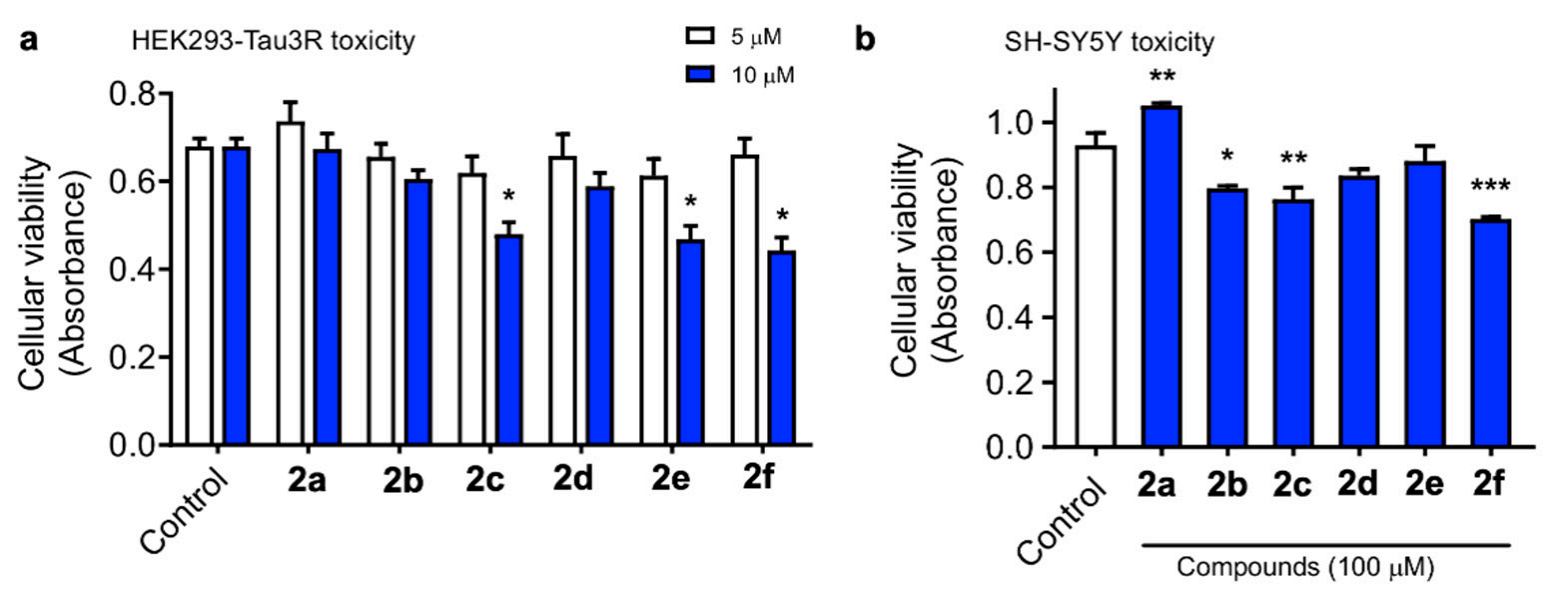
HEK293-Tau3R cells were treated with compounds **2** at 5 and 10 μM concentrations for 24 h (**a**). Similarly, SH-SH5Y cells were treated with the tested compounds at 100 μM concentration for 24 h (**b**). Cell viability was then measured by the MTT assay, and the data obtained are expressed as the mean ± S.E.M. of three independent experiments. Significant differences were found in the optical density (OD, 545 nm) values elicited by the addition of 10 μM **2c**, **2e** and the curcumin standard compared to the control untreated cells (Newman–Keuls post-hoc test, * *p* < 0.05 vs. control, ** *p* < 0.01 vs. control, *** *p* < 0.001 vs. control).

To start our biological evaluation, we first tested the potential toxic effect of curcumin/piperlongumine hybrids **2a**–**f**. We first employed HEK293-Tau3R cells, where human Tau has been overexpressed in human embryonic kidney 293 cells and constitute a good cell model to study Tau pathologies [53]. No increase in cell death was observed following treatment with a 5 μM concentration of compounds **2** up to 24 h. However, at 10 μM, some lowering of cell viability was observed with **2c**, **2e** and also with the curcumin standard (Figure 1a). Neurotoxicity was also evaluated in the SH-SY5Y neuronal cell line at a single, much higher, concentration (100 μM). As shown in Figure 1b, compounds **2b**, **2c** and **2f** showed significant toxicity; nevertheless, considering the high concentration used and the relatively low percentage of toxicity (31.4% viability reduction for the less favorable case, corresponding to **2f**), we consider these compounds to be safe.

#### 3.4.2. ROS Scavenger Activity in HEK293-Tau3R Cells

**Figure 2:**
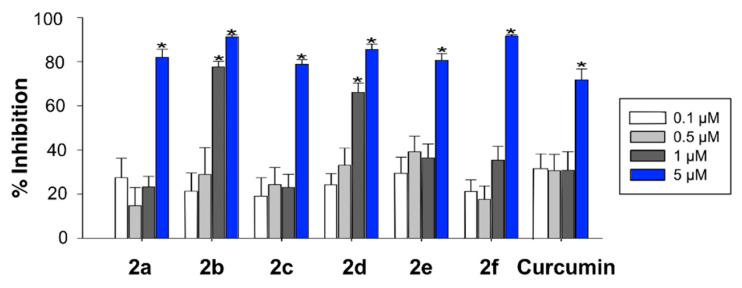
ROS scavenging activity of compounds **2** in HEK293-Tau3R cells, as measured by the inhibition of the ROS-promoted transformation of 2,7-dichlorofluorescin acetate into 2,7-dichlorofluorescein (Newman–Keuls post-hoc test, * *p* < 0.05 vs. control).

In order to complement the in vitro assays described above, radical oxygen scavenging of compounds **2** was tested with a cell-based antioxidant activity assay. To this end, HEK293-Tau3R cells were treated with hydrogen peroxide and with 2,7-dichlorofluorescin acetate (DCFA), which is trapped intracellularly as 2,7-dichlorofluorescin (DCFH) following its hydrolysis by cellular esterases. This intermediate, upon oxidation by ROS, is transformed into the highly fluorescent 2,7-dichlorofluorescein (DCF). Therefore, there is an inverse relationship between the fluorescence observed in the assay and the scavenging ability of the compounds. Exposure of HEK293-Tau3R cells to 200 μM hydrogen peroxide for 1 h caused an approximate 35-fold increase in fluorescence intensity, relative to untreated control cells. Treatment of the cells with compounds **2**, at several concentrations in the 0.1–5 μM range, diminished the fluorescence intensity in comparison to the cells exposed to hydrogen peroxide alone. Figure 2 summarizes the % of inhibition of the fluorescent emission for compounds **2**, showing that all of them were more potent than curcumin, in the order **2f** > **2b** > **2d** > **2a** ≈ **2e** > **2c** > curcumin, which is roughly parallel to the one found in the DPPH assay (**2e** > **2b** > **2d** > **2a** ≈ curcumin > **2f** > **2c**), although a perfect fit should not be expected due to the need for the compounds to cross a cell membrane in the case of the DCFA assay, which introduces membrane permeability as an additional factor.

#### 3.4.3. Nrf2 Induction

**Figure 3:**
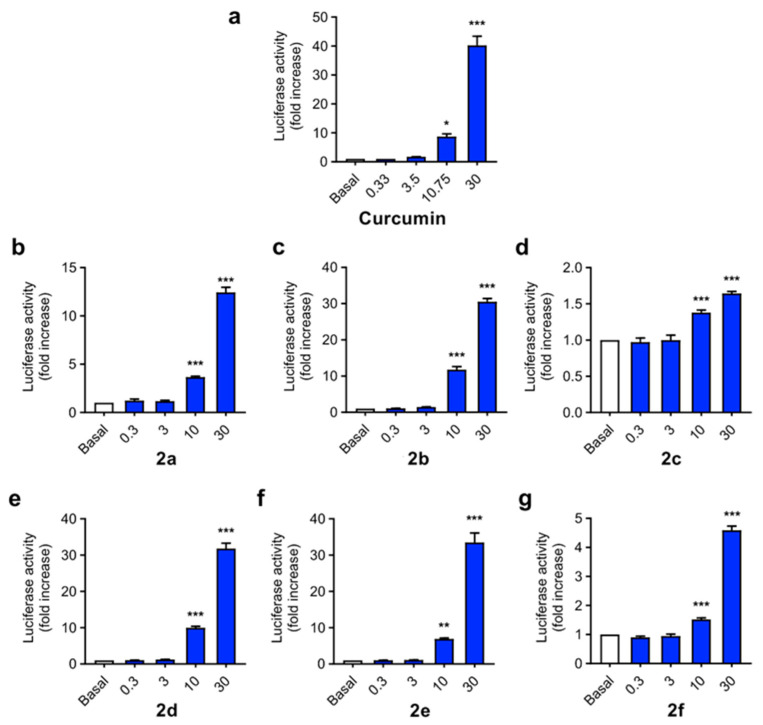
Phase II antioxidant response activation capacity of compounds **2a**–**f** compared to curcumin (the latter was shown to induce Nrf2 in [57]) in the AREc32 cell line. Cells were incubated with increasing concentrations of compounds (0.3, 3, 10 and 30 µM) or culture media during 24 h. Then, luciferase expression was tested by luminescence assay. Data are expressed as luciferase activity normalized to basal conditions considered as 1. Data are means ± SEM of four independent experiments in duplicate. One way ANOVA Newman Keuls post-hoc test: *** *p* < 0.001; ** *p* < 0.01; * *p* < 0.05 compared to the corresponding basal conditions.

As mentioned in the Introduction, curcumin is widely described as a Nrf2 inducer, being able to activate the phase II antioxidant response in different cellular types [54], including neuronal cells [55], and affording neuroprotection against Aβ and oxidative stress. Considering the design of our derivatives, we evaluated their capacity to activate the phase II antioxidant and anti-inflammatory response. To this end we used the AREc32 cell line, an MCF7 clone stably transfected with a luciferase gene construct under the control of eight copies of the rat Gsta2 ARE [56]. AREc32 cells were treated with four different concentrations (0.3, 3, 10 and 30 µM) of the compounds for 24 h; then, luciferase expression derived from Nrf2 activation was evaluated by a luminescence assay. Curcumin was previously described to activate Nrf2 response in the AREc32 cell line [57] (Figure 3a), showing a CD value (concentration needed to double luciferase expression) of 3.85 ± 0.15 µM. As expected, compounds **2a**–**f** were also able to induce Nrf2 (Figure 3b–g), with CD values ranging from 4.07 µM to 12.4 µM. Considering the substitution pattern, n-butyl derivatives showed higher potency as Nrf2 inducers than arylalkyl derivatives, with compound **2c** (R_1_ = F-Benzyl) showing the lowest activity. Alkyl derivatives **2b**, **2d** and **2e** showed similar potencies, being comparable to curcumin in terms of Nrf2 induction capacity, and the arylethyl derivative **2a** showed an intermediate potency. Modification of methyl carboxylic moiety (**2b)** to the corresponding ethyl derivative (**2d**) slightly decreased its potency, although the differences were non-significant. Modification of the aryl-allylidene moiety to include a phenolic ring, generating a ferulic acid-like moiety, also slightly decreased potency from CD = 4.7 ± 0.22 µM of compound **2a** to CD = 6.04 ± 0.21 µM of compound **2e**.

#### 3.4.4. Inhibition of PHF6 Aggregation

**Figure 4:**
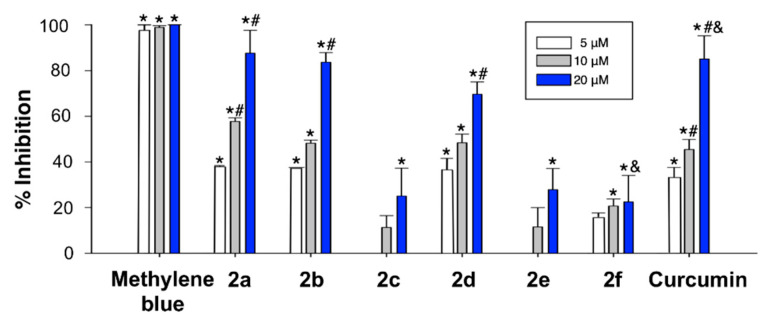
Plot showing the inhibition of aggregation of 100 µM PHF6 at the endpoint of ThT fluorescence set at 60 min. Effects of curcumin analogues on PHF6 aggregation after 60 min-incubation. The formation of fibrils was determined by the decrease in ThT fluorescence in the absence and presence of curcumin analogues. The samples were incubated at 37 °C, and either 100 μM of PHF6 in the absence and presence of 5–20 μM curcumin analogue. Experiments were performed in triplicate. Data are given as the mean lifetime ± SEM for each curcumin analogue concentration of three separate experiments. * Significant differences vs. 100 μM PHF6; # significant differences vs. 5 µM; & significant differences vs. 10 µM (*p* < 0.05, Newman-Keuls post-hoc test, in all cases).

The PHF6 (Ac-VQIVYK-NH2) Tau protein hexapeptide has been widely employed as a model for studying Tau aggregation. The kinetics of its aggregation following incubation in the presence or absence of the curcumin analogues **2** at several concentrations was monitored by the thioflavin T (ThT) fluorescence assay [58]. The fluorescence intensity of the ThT dye is in a direct relationship with the amount of amyloid formed in a solution. In our experiments, the PHF6 peptide (10 µM) started to aggregate immediately, without a lag phase, and in 60 min reached a plateau, showing completion of fibril formation (Figure 4). ThT fluorescence level showed a remarkable decline as the concentration of three of the curcumin analogues (**2a**, **2b** and **2d**) increased. Compounds **2c**, **2e** and **2f** also caused a decrease in the fluorescence intensity of PHF6, but to a significantly lesser extent, indicating a lower inhibitory capacity (Figure 4).

Among the screened compounds, **2a**, **2b** and **2d** were able to significantly reduce the aggregation propensity of PHF6 in a concentration dependent manner at sub-molar concentrations. These compounds might interact with the key residues of PHF6, which are responsible for β-sheet formation, and this is the likely mechanism of inhibition and disassembly.

**Figure 5:**
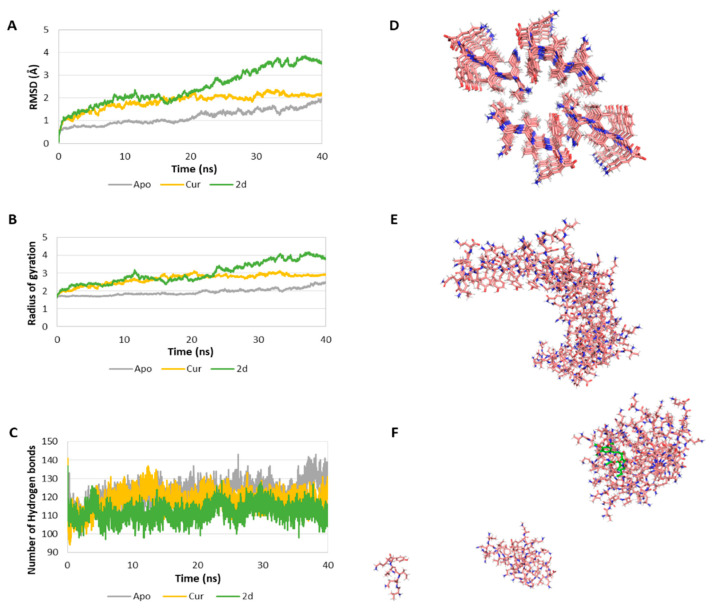
(**A**) RMSD of the Cα of the oligomer in the absence (Apo) and presence of curcumin (Cur) and compound **2d** as ligands. (**B**) Rg of the Cα of the oligomer in the absence and presence of ligand. (**C**) Number of H bonds among the monomers of the oligomer in the absence and presence of ligand. (**D**) First snapshot (t = 0 ns) of the Apo-system. In the system complexed with ligand, the ligand will be placed in the central hole. (**E**) Last snapshot (t = 40 ns) of the Apo system. (**F**) Last snapshot (40 ns) of the **2d**-complexed system. The ligand is coloured in green.

Due to the potential biological relevance of the interaction of our compounds with Tau protein, a computational study of the interaction of compounds **2** with the PHF6 peptide was undertaken in order to clarify their molecular inhibition mechanism at the atomic level in order to assist the development of more effective drugs to prevent Tau aggregation for the treatment of AD. These studies (Figure 5 and Table 4) were performed on the Tau protein hexapeptide (306–311, VQIVYK), widely employed in molecular dynamics [59,60], since Landau determined its experimental atomic coordinates [61]. This model consists of seven strands of four antiparallel β-sheets each (28 VQIVYK-monomer repeats). Our study started by evaluating the stability of the systems by calculating the root-mean-square deviation (RMSD) of the Cα of the oligomer in the absence of ligands (Apo-system) and in the presence of curcumin and compound **2d** (Figure 5A). This value is lowest (1.16 ± 0.33 Å) in the absence of ligand and increases in the presence or curcumin (1.85 ± 0.35 Å), and especially in the presence of **2d** (2.47 ± 0.80 Å). The ligand dependent-increase of the RMSD indicates a decrease of the stability of the system. The radius of gyration (Rg) of the Cα of the oligomer was also determined and found to increase significantly in the complexed systems in comparison with the Apo-system (Figure 5B), indicating a decrease in the compactness of the system upon ligand addition. We also determined the number of hydrogen bonds (Hbond) among monomers for all of the snapshots of the simulation as an estimation of the stability of the oligomer. The Apo-system showed the greatest number of hydrogen bonds (122.18 ± 6.13), while the curcumin-complexed oligomer showed a value of 118.23 ± 5.55, and in the presence of compound **2d** the Hbond parameter was even lower, 112.02 ± 4.70 (Figure 5C). A decrease in the number of hydrogen bonds among the monomers is another indication of a decrease in the stability of the oligomer in the presence of the ligands. In the aggregate, these results show that complexation with either curcumin or compound **2d** diminish the stability of the PHF6 oligomer, with compound **2d** exerting the most potent destabilization. A comparison of the system in the absence of ligands at t = 0 (Figure 5D), at t = 40 ns, showing aggregation (Figure 5E), and the same system in the presence of compound **2d** at the end of the 40 ns simulation clearly show the disruption of the aggregation process induced by the presence of our ligand.

**Figure 6:**
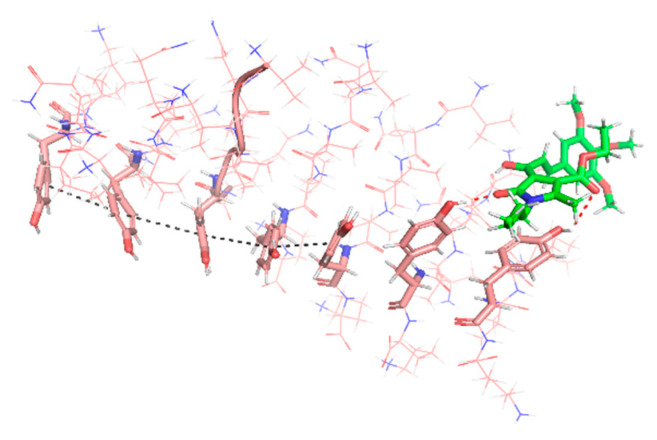
Compound **2d** (green) established two hydrogen bonds with the phenolic moieties of Tyr (red dashed lines), disordering the oligomer (pink). The highly ordered structure of one strand, stabilized by aromatic π–π stacking interactions (black dashed line), can be observed on the left-hand side of the image, while the orientation of the Tyr residues on the right-hand side is altered by their interaction with **2d**, hampering their stacking.

A major driving force of the self-association process of amyloidogenic proteins and the stabilization of the resulting fibrils is the π–π stacking interaction between the side chains of their aromatic amino acid residues [62]. In the case of many other small molecules able to inhibit PHF6 aggregation, interference in the protein–protein self-association comes from hydrophobic interactions of aromatic moieties of the compound with aromatic residues in the target protein [63,64]. In the case of compound **2d**, however, our results show that it interacts with its long axis perpendicular to the fiber axis via two hydrogen bonds involving two Tyr hydroxyls in the hexapeptide and the lactam and unsaturated ketone carbonyls in **2d**, plus an hydrophobic interactions of the n-butyl chain with the aromatic ring of a Tyr residue. These interactions cause a deviation of the Tyr residues and hamper their stacking (Figure 6).

#### 3.4.5. Anti-Inflammatory Properties

In normal conditions, microglia participates in the development of host brain defense and the maintenance of neuronal networks and tissue repair of central nervous system [65]. However, in AD microglia activity becomes detrimental and induces toxicity, in a process known as chronic neuroinflammation. Microglial activation is observed from the early stages of the disease, being considered as a potential actor in the onset and development of AD [66]. Aβ peptides interact with different microglial receptors, including TLR4 [67], CD36 [68] and NLRP3 [69], inducing their activation and promoting the liberation of pro-inflammatory factors such as TNFα and IL-1β that mediate neuroinflammatory response and exacerbate neuronal toxicity. In turn, increased neuroinflammation accelerates Aβ production and aggregation, accelerating the degenerative process. Considering the anti-inflammatory activity of both curcumin [10,11] and piperlongumine [20] and the antioxidant and Nrf2 induction capacity of derivatives **2**, we decided to evaluate their anti-inflammatory properties. To this end we used the BV2 microglial cell line and evaluated nitrite production stimulated by lipopolysaccharide (LPS). It is well known that LPS binding to TLRs activates the NF-κB signaling promoting the transcription of different pro-inflammatory genes such as the mentioned iNOS, NLRP3, and Pro-IL-1β, among others. Then, mature IL-1β also signals via TLRs to, again, activativate NF-κB [70]. The proinflammatory pathway activation increases iNOS enzyme levels that produces the proinflammatory mediator nitric oxide (NO) and, therefore, nitrite levels, its concentration being directly proportional to the activation of the pathway. LPS treatment promotes microglial cells polarization leading to an inflammatory state similar to disease conditions. Activated microglia express iNOS and nitric oxides, which are classically considered as polarization markers relevant for this inflammatory situation [71,72,73,74].

BV2 cells were treated with increasing concentrations of compounds **2a**–**f** (0.1, 0.3, 1 and 10 µM) for 24 h. This pre-incubation protocol was designed to allow the phase II antioxidant and anti-inflammatory response activation and the expression of Nrf2 regulated anti-inflammatory enzymes [75]. Thereafter, cells were co-incubated with compounds **2a**–**f** at the desired concentration and LPS (100 ng/mL) for 18 h. At the end of the co-incubation period, nitrite concentration was evaluated in supernatants of culture media by the Griess method. Curcumin was included as positive and reference compound (Table 5).

Interestingly, all derivatives showed a highly potent anti-inflammatory response with IC_50_s ranging from 0.30 ± 0.04 µM (compound **2a**) to 0.76 ± 0.13 µM (compound **2f**). With the exception of **2f**, all derivatives showed improved anti-inflammatory properties compared to curcumin (IC_50_ = 0.64 ± 0.17 µM). Interestingly, the nitrite reduction capacity showed only slight differences among compounds, suggesting that the anti-inflammatory capacity of compounds **2** is not directly correlated to any of the pharmacological properties previously described, appearing to be an effect related to their combination. As an example, compound **2c** (4-F-Bn derivative), showing the lowest Nrf2 induction capacity and the lowest scavenger effect in both ORAC and DPPH test, had an intermediate anti-inflammatory capacity (IC_50_ = 0.56 ± 0.17 µM), being slightly more potent than the curcumin although the differences were not significant.

#### 3.4.6. Neuroprotection against Oxidative Stress

Extensive oxidative stress is a key player in the neurodegeneration associated to AD [76]. It is observed from the early stages of the disease and it is proposed as part of the pathological status participating in the onset and development of AD. Exacerbated oxidative stress induces chronic neuroinflammation by directly activating glial cells [77] and inducing the proteinopathy observed in AD [76]. As demonstrated by our previously described studies, compounds **2a**–**f** exert antioxidant capacity, reduce ROS production in cells and induce Nrf2, a combination of activities that should prevent neuronal damage caused by high oxidative stress. Therefore, we set out to evaluate the potential neuroprotective properties of the curcumin/piperlongumine hybrids **2a**–**f** in a model of oxidative stress produced by mitochondrial toxicity, namely the rotenone-oligomycin A (R/O) toxic combination [78,79,80]. This combination inhibits complexes I and V of the mitochondrial respiratory chain, increasing the concentration of free radical species in the cytoplasm and inducing cellular death.

To carry out this study, we selected a pre-incubation protocol similar to the one employed for the evaluation of anti-inflammatory properties, in order to allow the expression of Nrf2-regulated antioxidant and neuroprotective enzymes. Thus, SH-SY5Y neuroblastoma cells were pre-treated with compounds **2** at a single concentration (1 µM) for 24 h. After this time, culture media was removed and cells were treated with the corresponding compound **2** and the R/O toxic mixture (30/10 µM respectively) for 24 h. Finally, cellular viability was assayed by the MTT method. Curcumin has shown neuroprotective activity in several studies with rotenone, some of which were performed in the same SH-SY5Y cell line [81,82], and similar results were reported in PC12 cells [83]. Rotenone is employed in our case in combination with oligomycin A, but both toxics target the electron transport chain of the mitochondria, thus leading to oxidative stress and similar cellular effects. The rotenone and oligomycin A mixture is used in another study in combination with LPS in primary mixed glial cultures, and under these conditions curcumin also leads to an increase in cell viability, recovering cells from the toxic condition [84].

**Figure 7:**
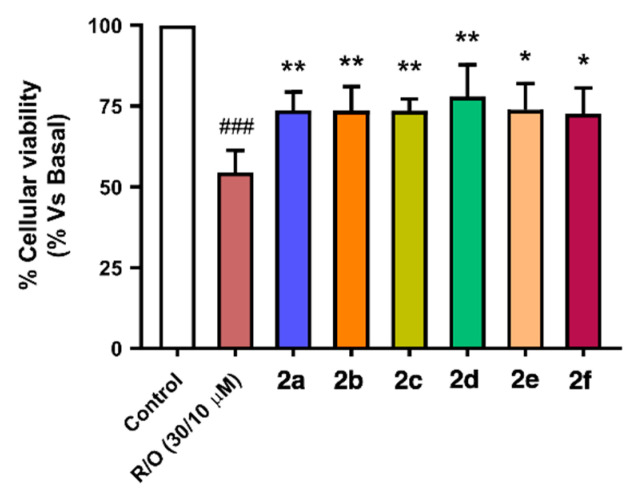
Neuroprotective capacity of compounds **2a**–**f** against oxidative stress induced by the toxic combination of rotenone (30 µM) and oligomycin A (10 µM). SH-SY5Y neuroblastoma cells were treated with the corresponding compound **2** (1 µM) for 24 h, then cells were co-incubated with the same compound and the R/O combination for further 24 h. Finally, cellular viability was assayed by the MTT method. Data are means of six different cultures in duplicate. One way ANOVA Newman Keuls post-hoc test: ^###^ *p* < 0.001 compared to basal; * *p* < 0.05, ** *p* < 0.01 compared to R/O.

As shown in Figure 7, compounds **2a**–**f** showed neuroprotective capacity towards oxidative stress, and it is interesting to note that aromatic substituted derivatives **2a**, **2c** and **2f** were less potent than alkyl derivatives **2b**, **2d** and **2e**, being derivative **2d** (^n^Bu, ethyl ester) the most potent neuroprotective agent with a 61% protection. Interestingly, this activity correlates well with their Nrf2 induction capacity and anti-oxidant properties, both in ORAC and DPPH assays considering the combination of them. For example, compound **2d**, the most potent neuroprotectant, is also the second most potent Nrf2 inducer (CD = 4.90 ± 0.21 µM), a potent scavenger in the ORAC assay (1.63 ± 0.06 trolox equivalents, T.Eq.) and the third most potent compound in the DPPH assay (SC_50_ = 14.6 ± 3.17 µM). The second most potent neuroprotectant derivative **2e** (^n^Bu, -OH and -OMe aromatic substituents, 47.9% protection) showed poorer Nrf2 induction capacity (CD = 6.04 ± 0.21 µM) combined with the most potent antioxidant capacity at the ORAC (1.73 ± 0.68 T.Eq.) and DPPH assays (SC_50_ = 8.87 ± 0.45 µM). Finally, the most potent Nrf2 inducer (CD = 4.07 ± 0.22 µM), compound **2b** (^n^Bu), presented the third most potent neuroprotective capacity, being a poor scavenger in the ORAC assay (0.52 ± 0.22 T.Eq.) and the intermediate scavenger in the DPPH assay (SC_50_ = 10.8 ± 0.06 µM). These results demonstrate that the neuroprotective activity of compounds **2a**–**f** against exacerbated oxidative stress depends on the combination of pharmacological properties.

#### 3.4.7. Neuroprotection against Tau Hyperphosphorylation Induced by Okadaic Acid

As previously described, AD is characterized by the formation of PHF and NFT aberrant aggregates of Tau protein. Tau aggregates are able to induce neurotoxicity by different mechanisms of action including increasing oxidative stress and chronic neuroinflammation [85]. Moreover, exacerbated oxidative stress accelerates Tau hyperphosphorylation and aggregation, generating a feedback positive loop that accelerates neurodegeneration [86]. In fact, the formation of hyperphosphorylated Tau aggregates has been demonstrated to correlate with the cognitive decline observed in advanced phases of AD.

In addition to the Nrf2 induction capacity, antioxidant and anti-inflammatory properties of compounds **2a**–**f**, we have demonstrated their PHF6 aggregation inhibitory capacity, a property of high interest for AD treatment. Therefore, the multitarget combination of these compounds prompted the evaluation of their potential neuroprotective capacity against the toxicity induced by Tau hyperphosphorylation/aggregation. We used the well-characterized okadaic acid (OA) model of Tau hyperphosphorylation and neurotoxicity [87,88]. OA is a protein phosphatase 2A inhibitor able to promote Tau hyperphosphorylation and neurotoxicity [23,89]. Curcumin has been shown to exert a neuroprotective effect on okadaic acid-induced memory impairment in mice. In this study, curcumin reduced ROS and nitrites and increased GSH in the mice cortex and hippocampus, among other effects in different behavioral tests and biochemical parameters [90].

**Figure 8:**
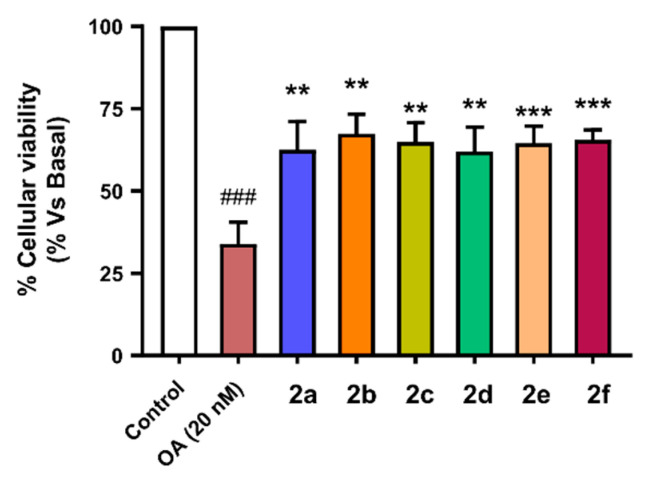
Neuroprotective capacity of compounds **2a**–**f** against Tau hyperphosphorylation induced by treatment with okadaic acid (OA, 20 nM). SH-SY5Y neuroblastoma cells were treated with the corresponding compound (1 µM) during 24 h, and then cells were co-incubated with the corresponding compound and the OA (20 nM) combination for a further 24 h. Finally, cellular viability was assayed by the MTT method. Data are the means of six different cultures in duplicate. One way ANOVA Newman Keuls post-hoc test: ^###^ *p* < 0.001 compared to basal; ** *p* < 0.01, *** *p* < 0.001 compared to OA.

To evaluate the neuroprotective capacity of compounds **2a**–**f** we selected also the SH-SY5Y cellular line using the same pre-incubation and co-incubation protocol used in previous experiments. Cells were incubated with the corresponding compounds at 1 µM concentration during 24 h. Thereafter, they were co-incubated with the corresponding compound (1 µM) and OA (20 nM) for 24 h. Finally, cellular viability was assessed by the MTT method. Successfully, compounds **2a**–**f** showed good neuroprotective activity against toxicity induced by OA with protection percentages ranging from 55.6% protection of compound **2b** (^n^Bu-) to 39.1% protection shown by derivative **2e** (^n^Bu, -OH, -OMe) (Figure 8). There is a clear correlation between the PHF6 aggregation inhibitory capacity of derivatives **2a**–**f** and their respective neuroprotective capacity. Previously, we described compounds **2a**, **2b** and **2d** as the most potent PHF6 aggregation inhibitors and their neuroprotective capacities against OA-induced Tau hyperphosphorylation were 50.0% (**2a**), 55.6% (**2b**), and 48.6% (**2d**). The most potent neuroprotection was exerted by compound **2b**, which was also the most potent Nrf2 inducer (CD = 4.07 ± 0.22 µM).

## 4. Conclusions

The antioxidant, anti-inflammatory and protein anti-aggregating properties of curcumin render this natural product a good starting point for the discovery of pharmacological treatments of neurodegenerative diseases. Unfortunately, curcumin has serious drawbacks arising from its poor membrane permeability and its low chemical and metabolic stability. Curcumin-like compounds were designed by hybridization of its structure with that of the natural neuroinflammation inhibitor piperlongumine, and were predicted computationally to have good oral absorption and CNS permeation. Although endowed with some structural complexity, these molecules were readily available via a two-step route and their pharmacological study uncovered good scavenging properties, both in in vitro and cellular assays, and potent induction of the phase II antioxidant response associated to the Nrf2-ARE pathway. Besides this promising antioxidant profile, the compounds inhibited the aggregation of the Tau PHF6 peptide and showed activity against the LPS-induced inflammatory response. Finally, the compounds were neuroprotective against oxidative stress induced by treatment with rotenone-oligomycin and also against Tau hyperphosphorylation induced by okadaic acid. This multitarget pharmacological profile constitutes a good starting point for future optimization efforts in the area of AD disease treatment.

## Figures and Tables

**Table 1:** Physicochemical and ADME properties predicted for compounds **2a**–**f**.

Cmpd.	MW (g/mol)	TPSA (Å^2^)	Heavy Atoms	HBA	HBD	RotB	Log P	QPP Caco (nm/s)	QPP MDCK (nm/s)	%Human Oral Absorption	Rule of Five
**Curcumin**	368.38	96.22	27	6	3	12	3.17	141.2	59.6	81.8	0
**2a**	497.51	94.53	36	8	1	12	4.09	903.3	799.9	100	1
**2b**	431.48	94.53	31	7	1	12	3.13	864.1	422.5	96.5	1
**2c**	483.49	94.53	35	8	1	11	3.71	428.8	357.5	95.8	1
**2d**	445.51	94.53	32	7	1	13	3.47	1044.3	518.4	100	1
**2e**	387.43	96.3	28	6	2	11	2.69	314.4	141.6	100	0
**2f**	465.50	94.53	34	7	1	11	3.36	980.3	484.2	100	1

MW: molecular weight; TPSA: topological polar surface area; HBA: number of hydrogen bond acceptors; HBD: number of hydrogen bond donors; RotB: Number of non-trivial (not CX3), non-hindered (not alkene, amide, small ring) rotatable bonds; log P: Octanol/water partition coefficient; QPP Caco: Predicted apparent Caco-2 cell permeability; QPP MDCK: Predicted apparent MDCK cell permeability; Percent Human Oral Absorption: Predicted human oral absorption on 0 to 100% scale; Rule of five: Number of violations of Lipinski’s rule of five (one violation is allowed).

**Table 2:** Predicted CNS permeability of compounds **2**.

Entry	Compound	CNS MPO.v2 [0–6]
1	**Curcumin**	3.97
2	**2a**	4.03
3	**2b**	4.93
4	**2c**	4.23
5	**2d**	4.63
6	**2e**	4.60
7	**2f**	4.53

**Table 3:** Antioxidant activity of compounds **2a**–**f** at the ORAC and DPPH assays ^a^.

Entry	Compound	ORAC	DPPH
		Trolox Eqs.	SC_50_, μM
1	**Curcumin**	3.88 ± 0.18	18.2 ± 0.63
2	**Ascorbic acid**	--	17.2 ± 1.14
3	**2a**	0.50 ± 0.23 ***	20.6 ± 2.17
4	**2b**	0.52 ± 0.22 ***	10.8 ± 0.06 ***/^###^
5	**2c**	0.35 ± 0.13 ***	56.9 ± 1.18 **/^##^
6	**2d**	1.63 ± 0.06 ***	14.2 ± 2.15
7	**2e**	1.73 ± 0.68 ***	8.87 ± 0.45 ***/^###^
8	**2f**	0.65 ± 0.11 ***	36.6 ± 1.02

^a^ Data are expressed as means ± SEM of five different experiments in duplicate. ** *p* < 0.01; *** *p* < 0.001 compared to curcumin. ^##^ *p* < 0.01; ^###^ *p* < 0.001 compared to ascorbic acid.

**Table 4:** Summary of the structural parameters determined for each system.

Parameter	Apo-System	With Curcumin	With Compound 2d
RMSD (Å)	1.16 ± 0.33	1.85 ± 0.35	2.47 ± 0.80
Rg	1.94 ± 0.19	2.33 ± 0.24	3.00 ± 0.60
Hbond	122.18 ± 6.13	118.23 ± 5.55	112.02 ± 4.70

**Table 5:** Reduction in nitrite production afforded by derivatives **2a**–**f** in BV2 cells stimulated by LPS. BV2 cells were pre-treated with the compound under assay at increasing concentrations (0.1, 0.3, 1 and 10 μM) for 24 h, then medium was replaced by fresh one containing compounds and LPS (100 ng/mL) for 18 h. The production of nitrite was assessed by the Griess method. Data shown in the Table are the mean of four different experiments, carried out in duplicate.

Entry	Compound	IC_50_ (μM) BV2
1	**Curcumin**	0.64 ± 0.17
2	**2a**	0.30 ± 0.04 *
3	**2b**	0.42 ± 0.08
4	**2c**	0.56 ± 0.03
5	**2d**	0.55 ± 0.08
6	**2e**	0.37 ± 0.05
7	**2f**	0.76 ± 0.13

Data are expressed as means ± SEM of four different experiments in duplicate. * *p* < 0.05 compared to curcumin.

## Data Availability

Data are contained within the article or Supplementary Materials.

## Supplementary Materials

The following are available online at https://www.mdpi.com/article/10.3390/antiox11010028/s1: Copies of spectra of new compounds.
