# Supplementary material for: Curcumin-Piperlongumine Hybrids with a Multitarget Profile Elicit Neuroprotection in In Vitro Models of Oxidative Stress and Hyperphosphorylation
Source: Antioxidants (Basel). 2021 Dec 24;11(1):28. doi: 10.3390/antiox11010028 (PMC8773050; doi:10.3390/antiox11010028)

Supporting Information

Multitarget curcumin-piperlongumine hybrids with antioxidant and antiinflammatory properties for the potential treatment of Alzheimer's disease.

Ángel Cores[1], Noelia Carmona-Zafra[1], Olmo Martín-Cámara[1], Juan Domingo Sánchez[1,2], Pablo Duarte[2,3], Mercedes Villacampa[1], Paloma Bermejo-Bescós[4], Sagrario Martín-Aragón[4*], Rafael León[2*] and J. Carlos Menéndez[1*]

[1] Unidad de Química Orgánica y Farmacéutica, Departamento de Química en Ciencias Farmacéuticas, Facultad de Farmacia, Universidad Complutense, 28040 Madrid, Spain.
[2] Instituto de Química Médica, Consejo Superior de Investigaciones Científicas (IQM-CSIC), 28006 Madrid, Spain.
[3] Instituto Teófilo Hernando y Departamento de Bioquímica, Facultad de Medicina, Instituto de Investigaciones Biomédicas 'Alberto Sols' UAM-CSIC, Universidad Autónoma de Madrid, Madrid, Spain.
[4] Departamento de Farmacología, Farmacognosia y Botánica, Facultad de Farmacia, Universidad Complutense, 28040 Madrid, Spain.

Copies of spectra of new compounds

Methyl 2-methyl-5-oxo-1-(4-flourophenethyl)-4,5-dihydropyrrole-3-carboxylate (**1a**)

^{1}H NMR (250 MHz, $CDCl_3$)

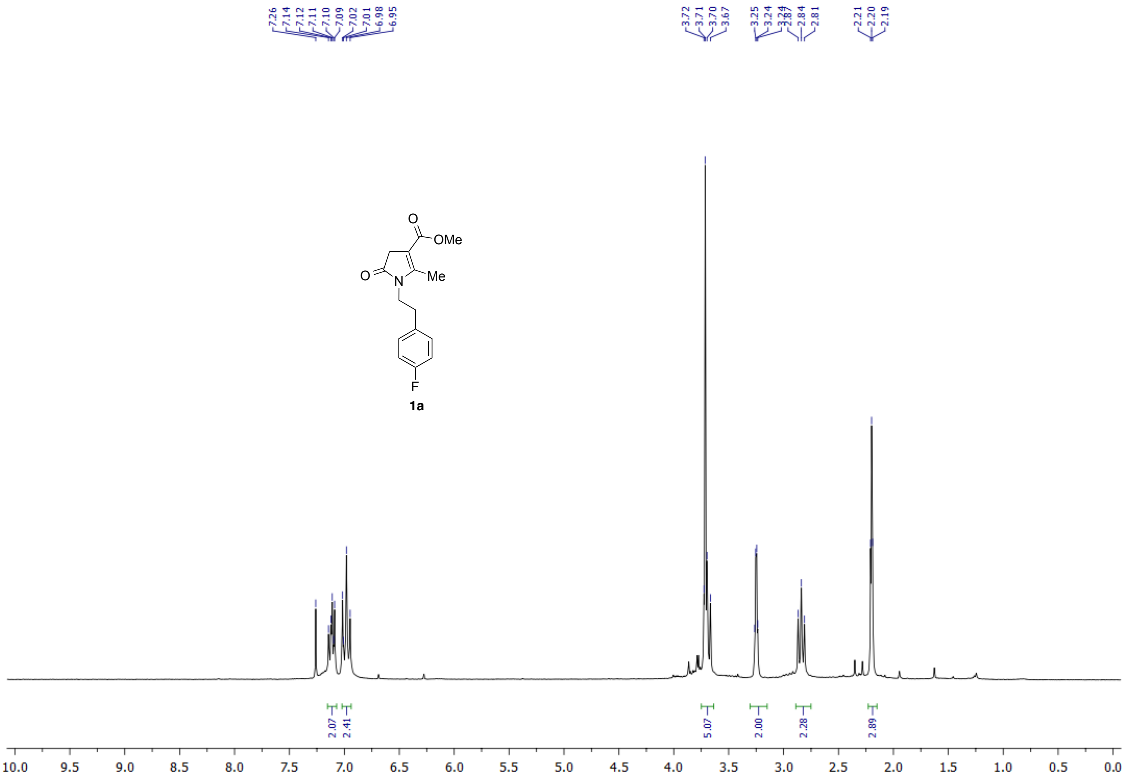

^{13}C NMR (63 MHz, $CDCl_3$)

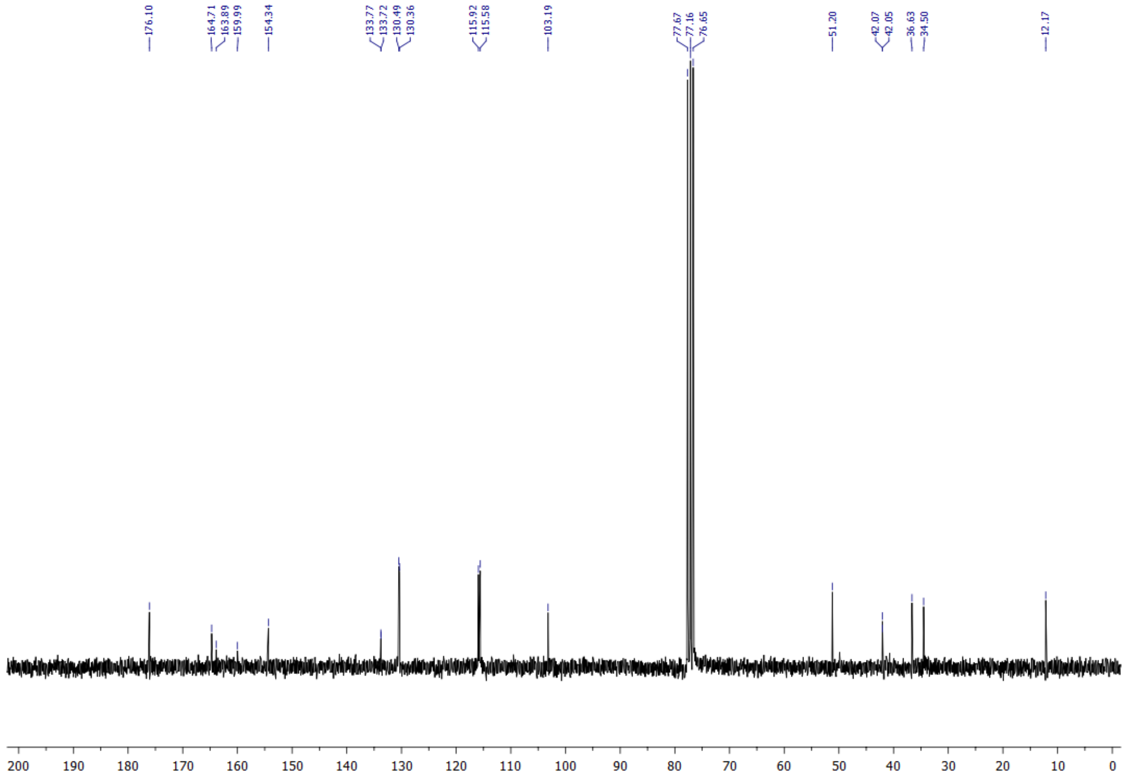

Methyl 1-(4-fluorobenzyl)-2-methyl-5-oxo-4,5-dihydropyrrole-3-carboxylate (**1c**)

^{1}H NMR (250 MHz, $CDCl_3$)

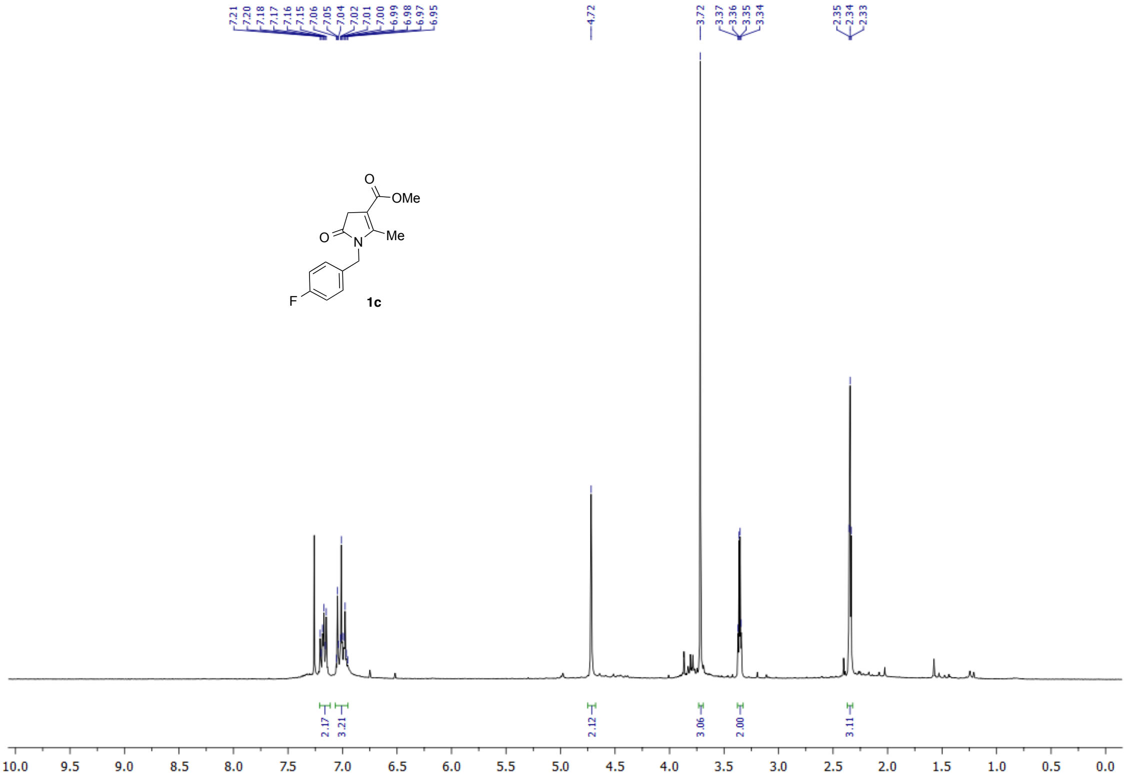

^{13}C NMR (63 MHz, $CDCl_3$)

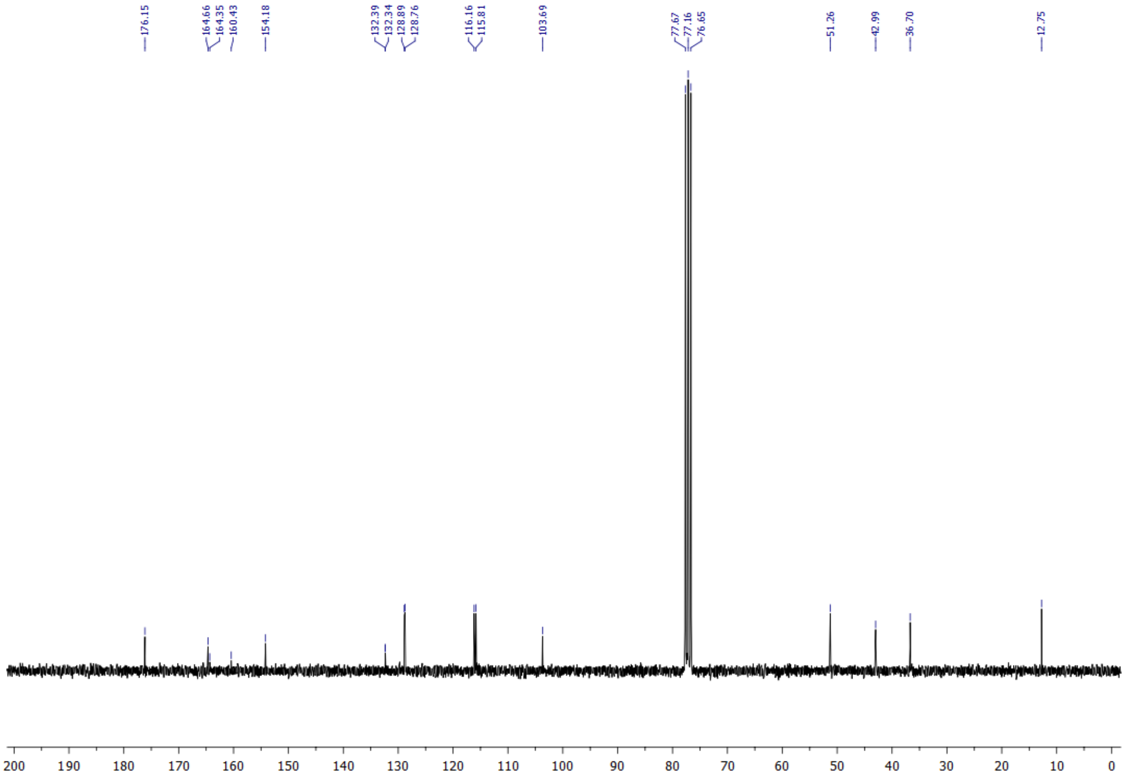

Methyl (*Z*)-1-(4-fluorophenethyl)-4-((*E*)-1-hydroxy-3-(3,4,5-trimethoxyphenyl)-allylidene)-2-methyl-5-oxo-4,5-dihydro-1*H*-pyrrole-3-carboxylate (**2a**)

^{1}H NMR (250 MHz, CDCl$_3$)

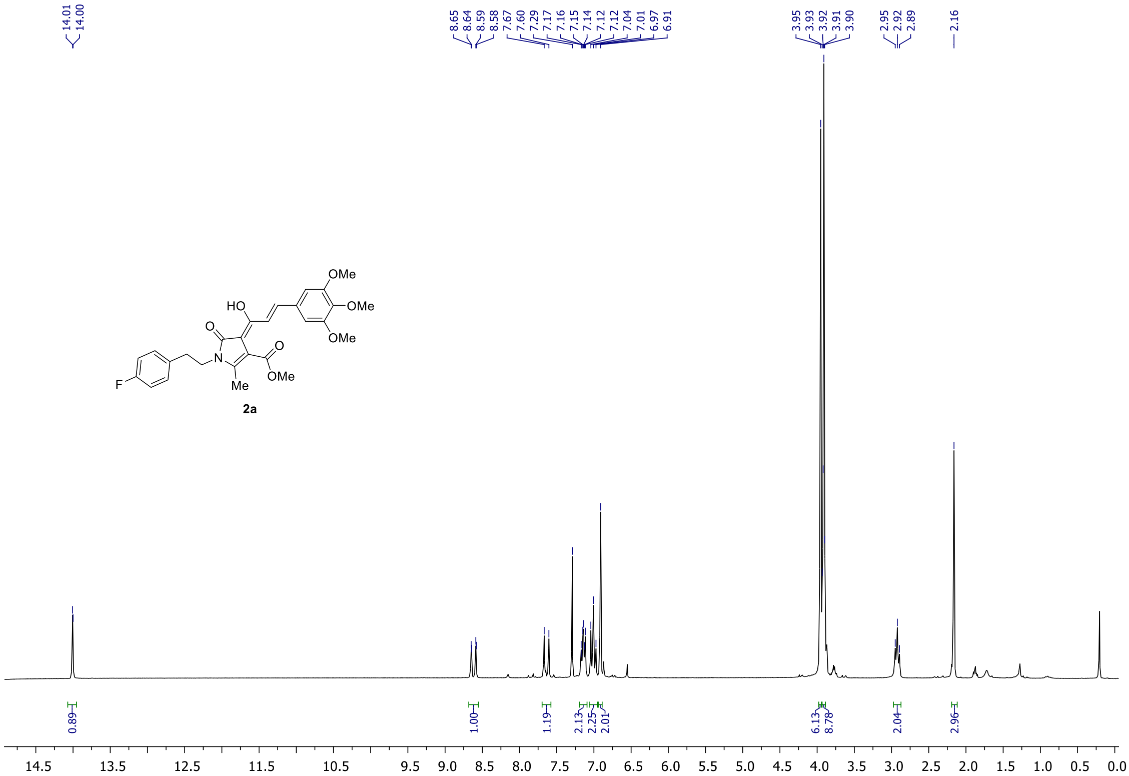

^{13}C NMR (63 MHz, CDCl$_3$)

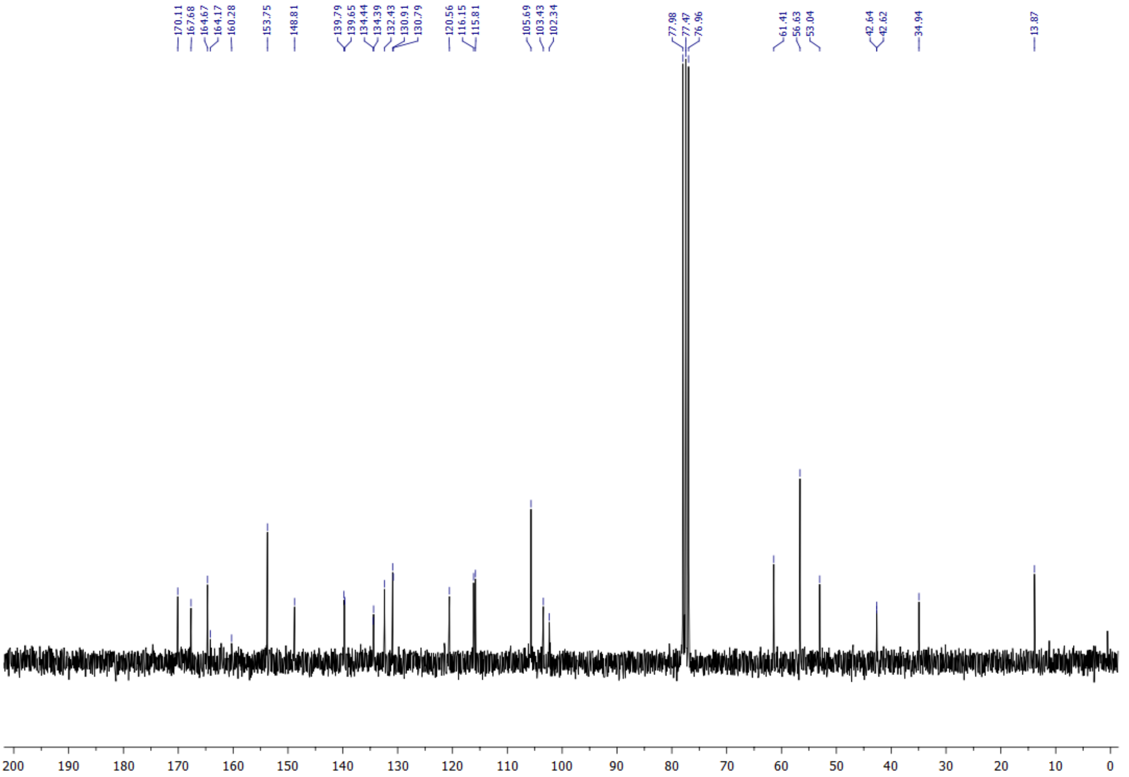

Methyl (*Z*)-1-butyl-4-((*E*)-1-hydroxy-3-(3,4,5-trimethoxyphenyl)allylidene)- 2-methyl-5-oxo-4,5-dihydro-1*H*-pyrrole-3-carboxylate (**2b**)

^{1}H NMR (250 MHz, CDCl$_3$)

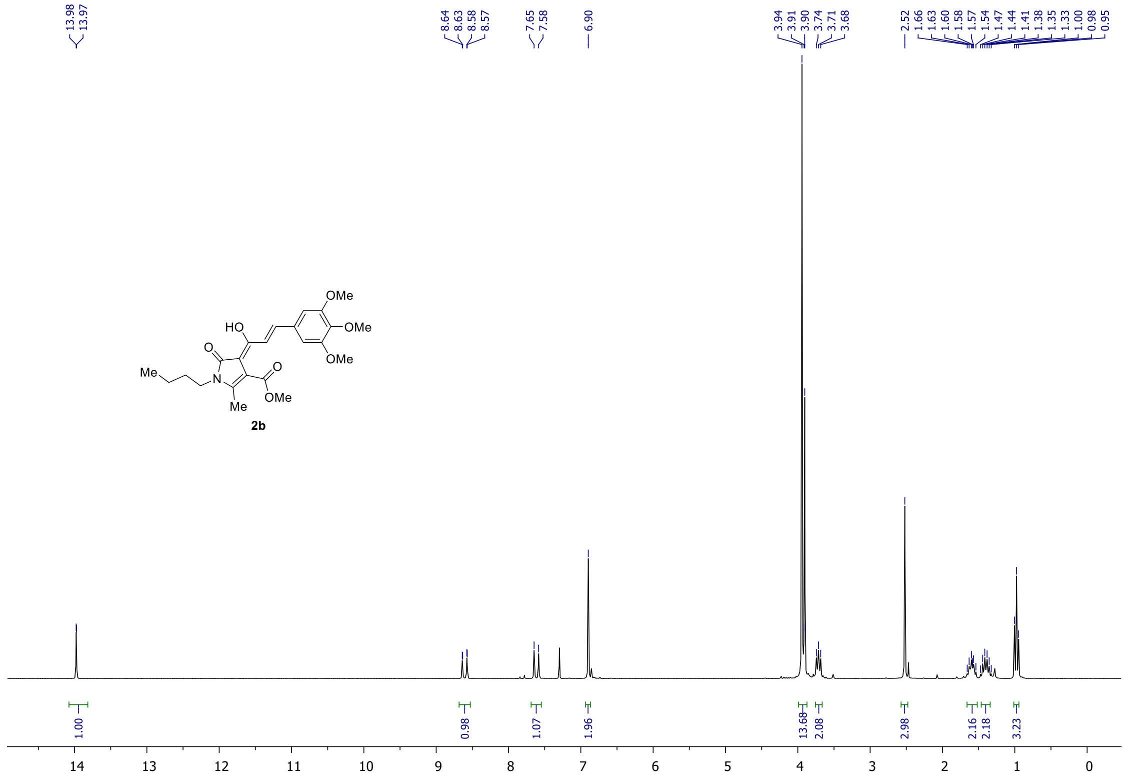

^{13}C NMR (63 MHz, CDCl$_3$)

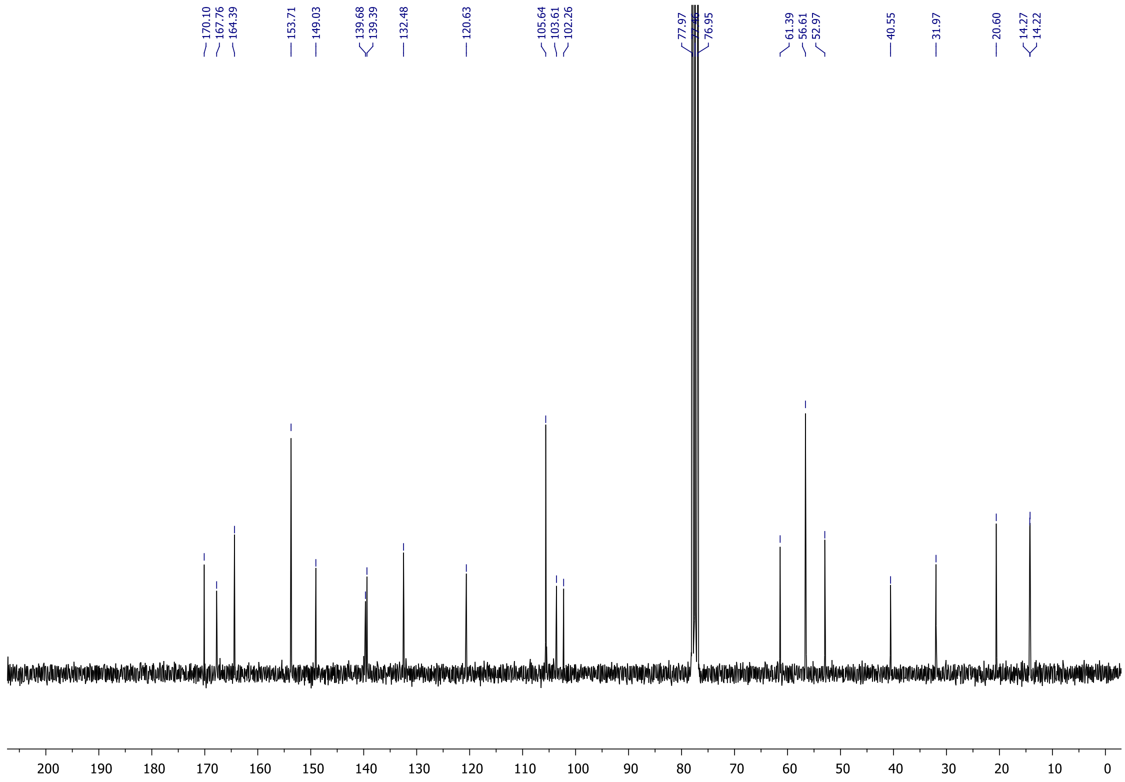

(*Z*)-1-(4-fluorobenzyl)-4-((*E*)-1-hydroxy-3-(3,4,5-trimethoxyphenyl)allylidene)-2-methyl-5-oxo-4,5-dihydro-1*H*-pyrrole-3-carboxylate (**2c**)

^{1}H NMR (250 MHz, $CDCl_3$)

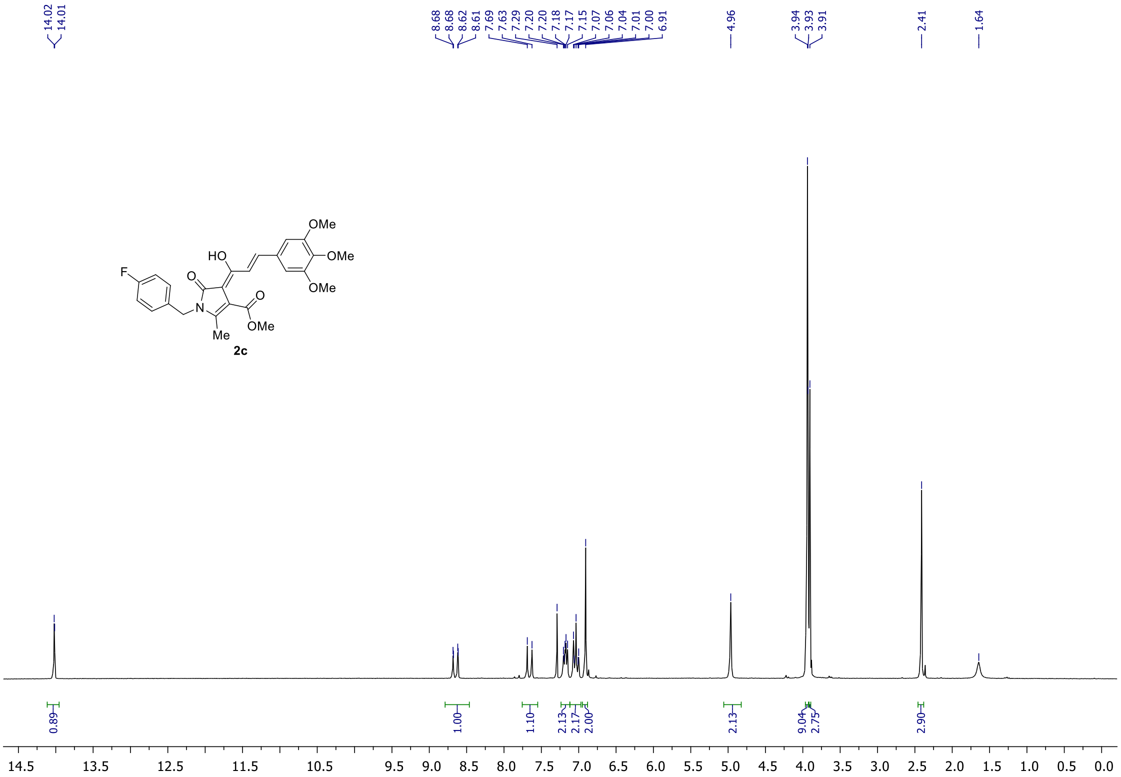

^{13}C NMR (63 MHz, $CDCl_3$)

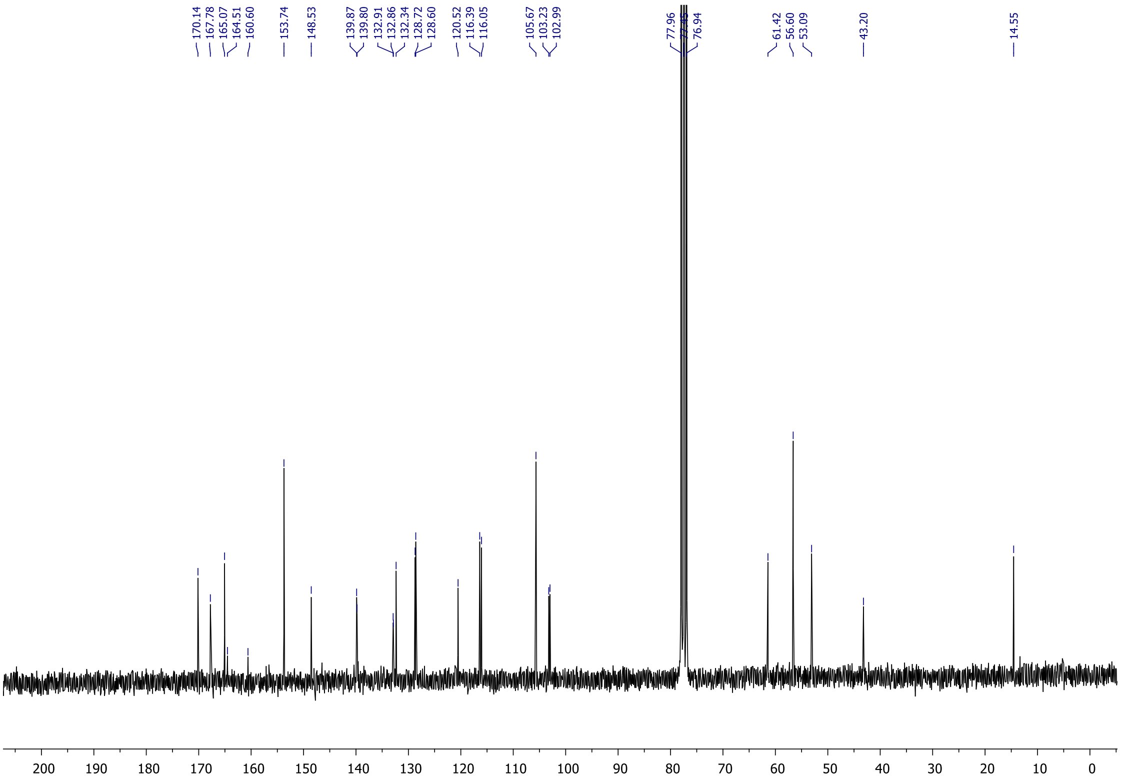

Ethyl (*Z*)-1-butyl-4-((*E*)-1-hydroxy-3-(3,4,5-trimethoxyphenyl)allylidene)- 2-methyl-5-oxo-4,5-dihydro-1*H*-pyrrole-3-carboxylate (**2d**)

^{1}H NMR (250 MHz, $CDCl_3$)

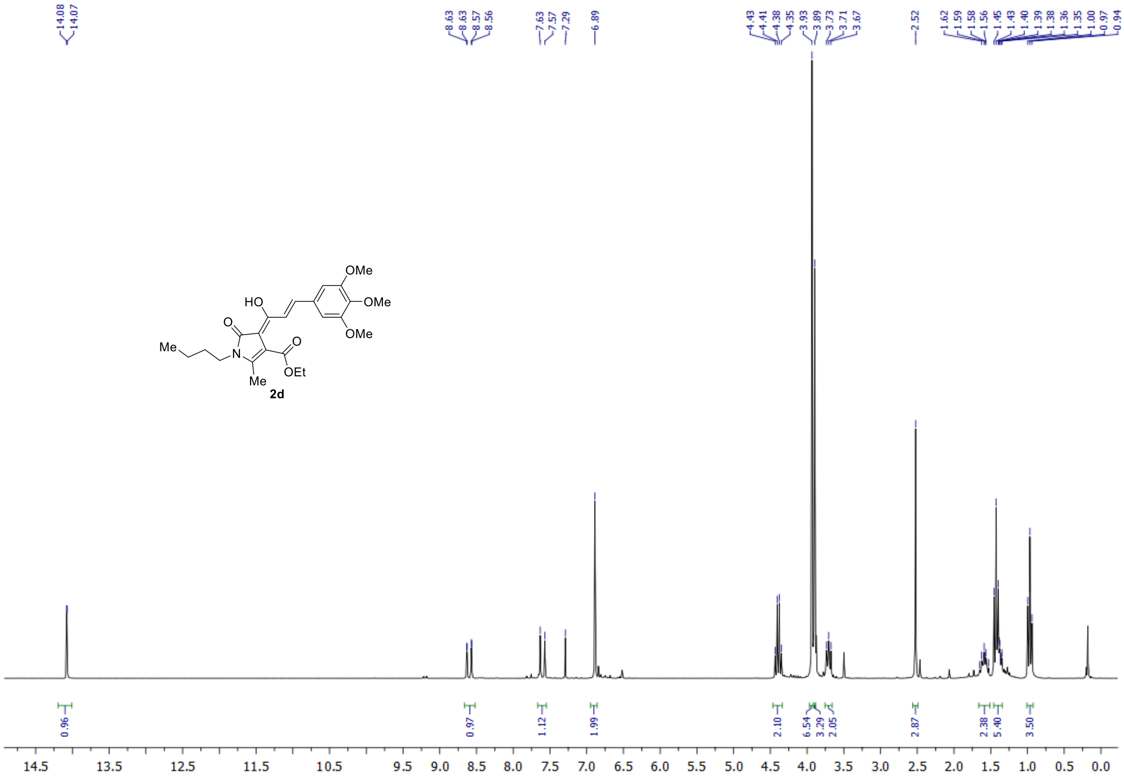

^{13}C NMR (63 MHz, $CDCl_3$)

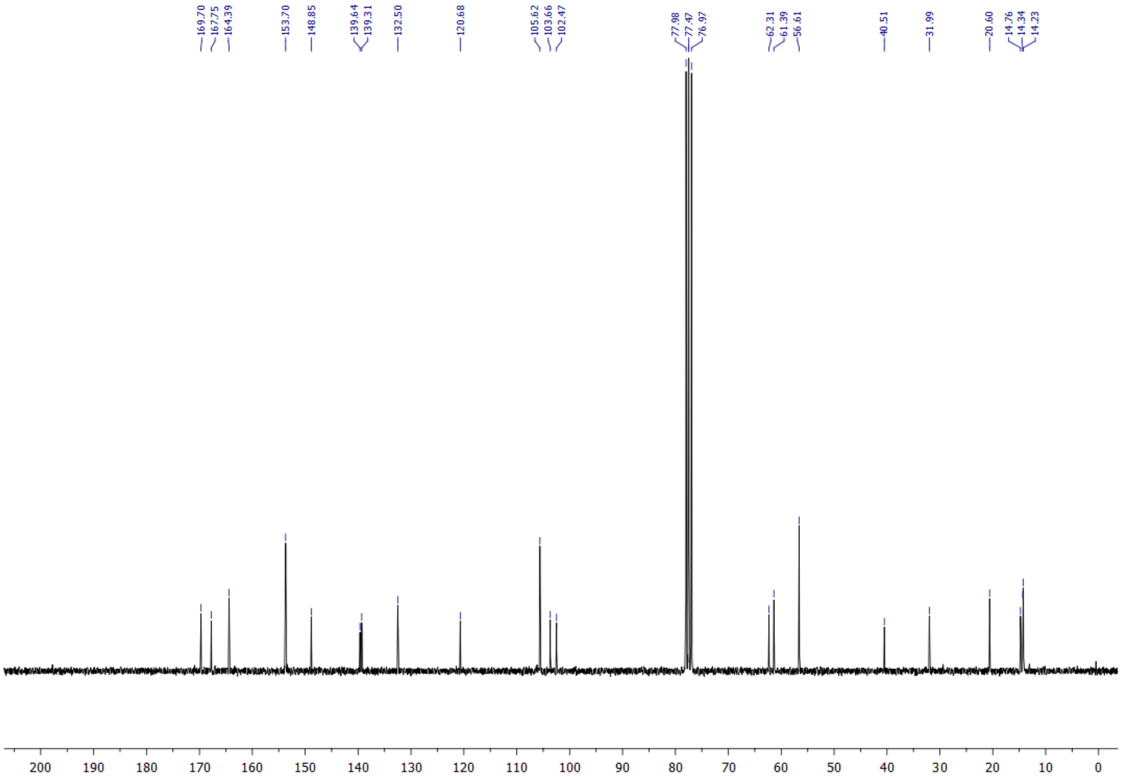

Methyl (*Z*)-1-butyl-4-((*E*)-1-hydroxy-3-(4-hydroxy-3-methoxyphenyl)allylidene)- 2-methyl-5-oxo-4,5-dihydro-1*H*-pyrrole-3-carboxylate (**2e**)

^{1}H NMR (250 MHz, CDCl$_3$)

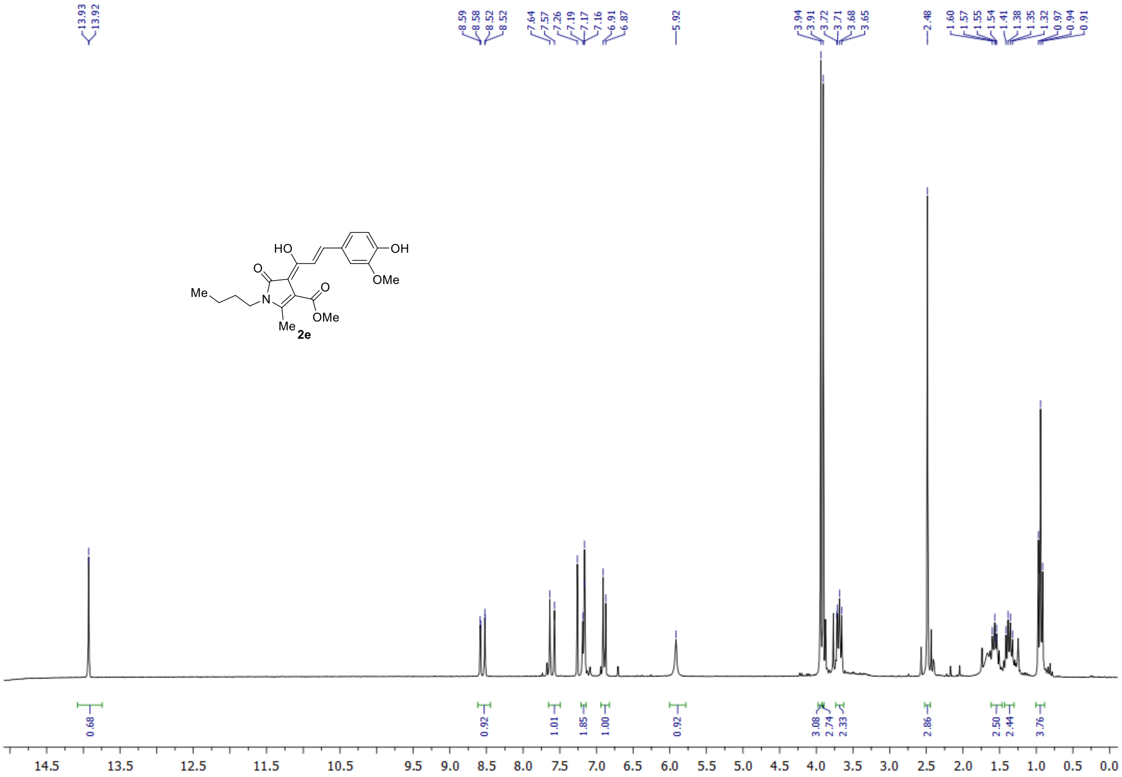

^{13}C NMR (63 MHz, CDCl$_3$)

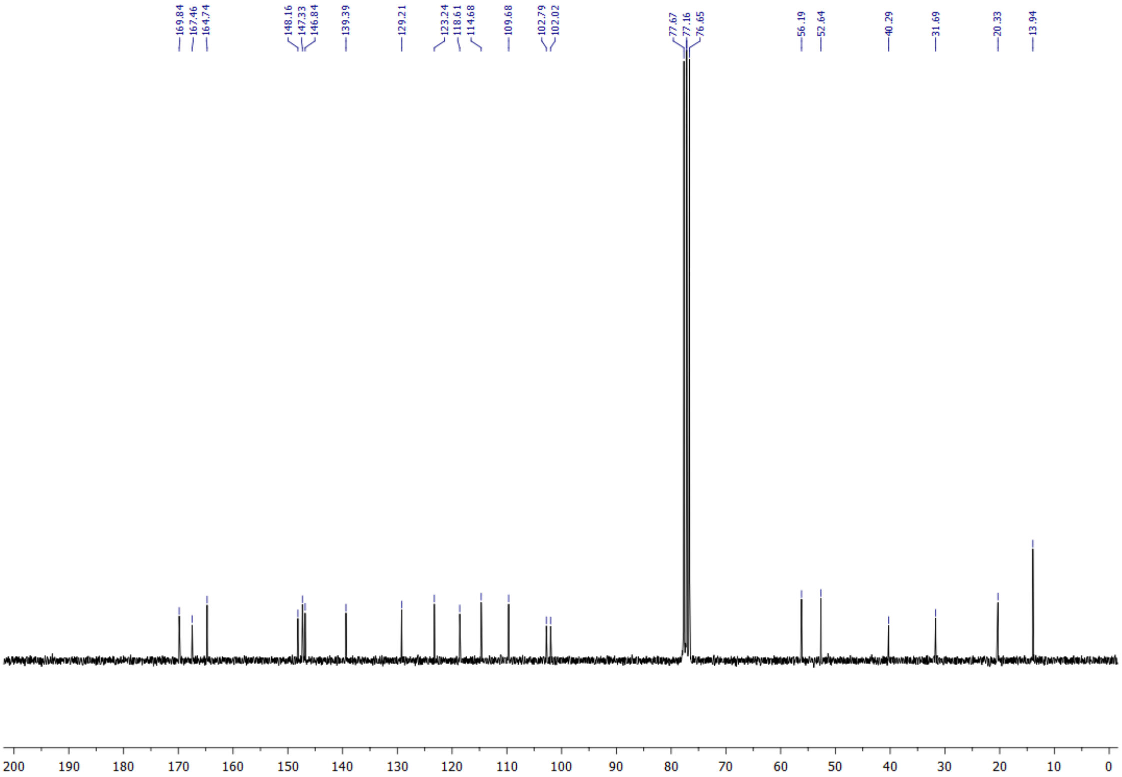

Methyl (*Z*)-1-benzyl-4-((*E*)-1-hydroxy-3-(3,4,5-trimethoxyphenyl)allylidene)- 2-methyl-5-oxo-4,5-dihydro-1*H*-pyrrole-3-carboxylate (**2f**)

^{1}H NMR (250 MHz, CDCl$_3$)

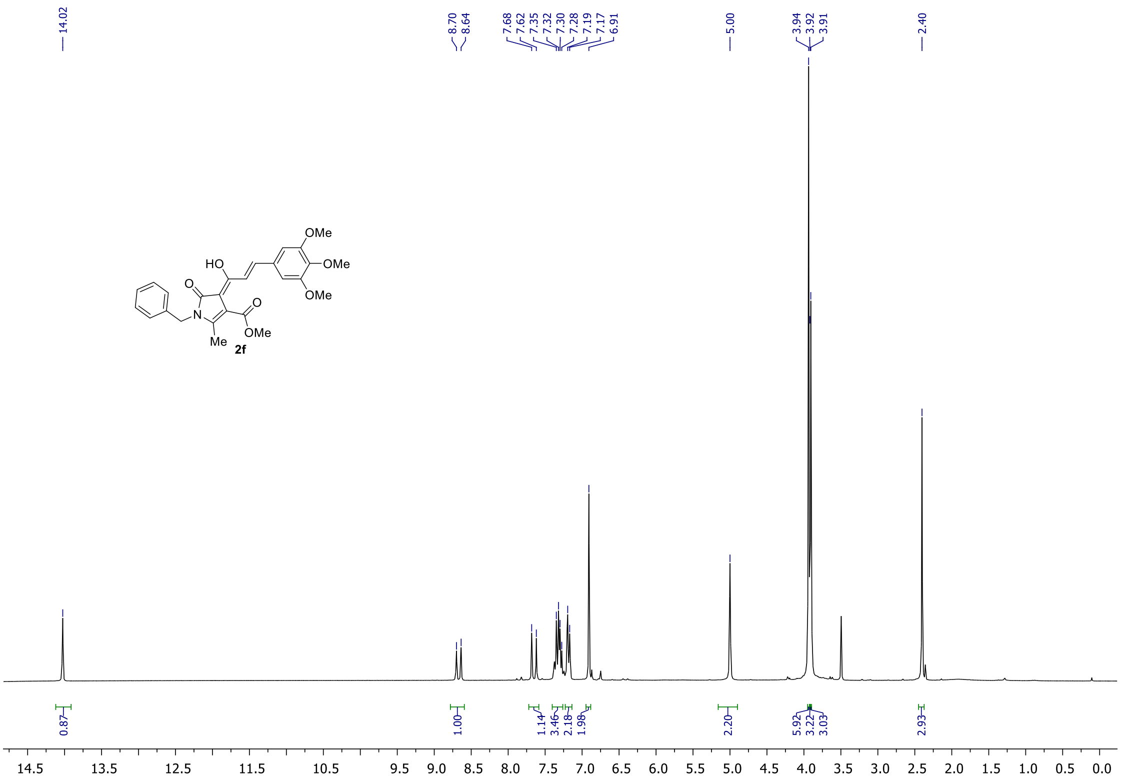

^{13}C NMR (63 MHz, CDCl$_3$)

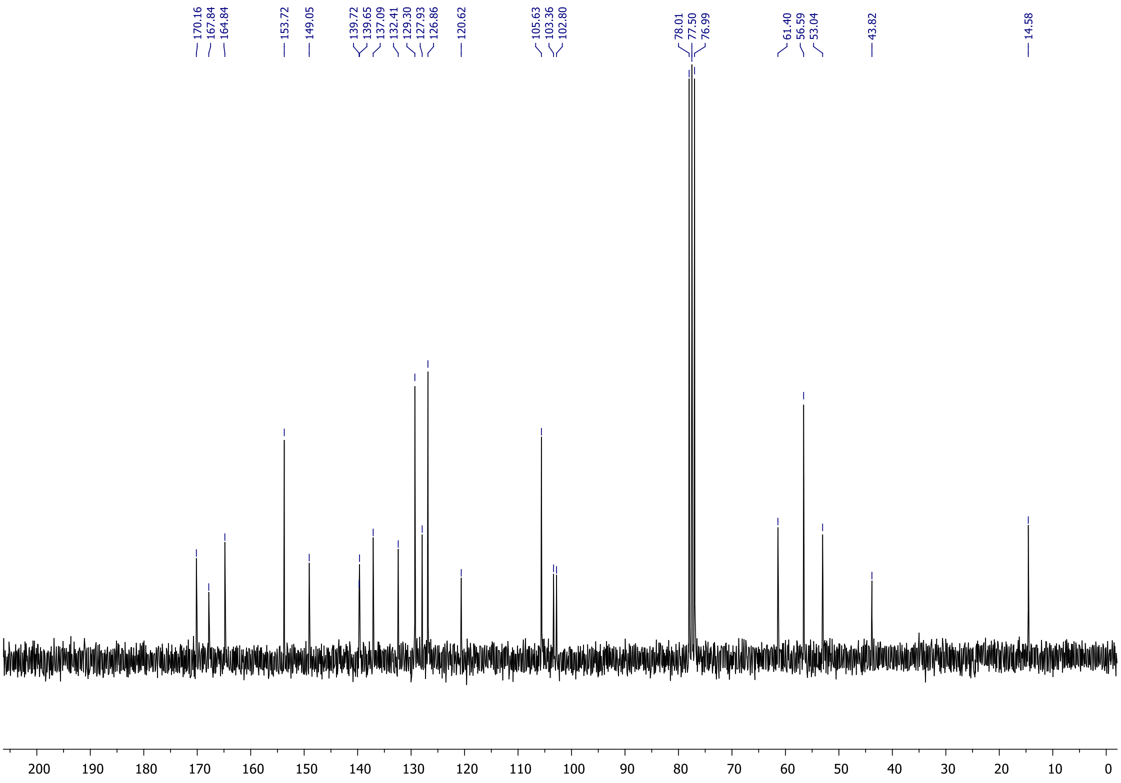

Supplement: Supplementary file 1 [file antioxidants-11-00028-s001.zip › antioxidants-1497081-supplementary.pdf]
